# Application of Computational Data Modeling to a Large-Scale Population Cohort Assists the Discovery of Inositol as a Strain-Specific Substrate for *Faecalibacterium prausnitzii*

**DOI:** 10.3390/nu15061311

**Published:** 2023-03-07

**Authors:** Shaillay Kumar Dogra, Adrien Dardinier, Fabio Mainardi, Léa Siegwald, Simona Bartova, Caroline Le Roy, Chieh Jason Chou

**Affiliations:** 1 Department of Gastrointestinal Health, Nestlé Institute of Health Sciences, Nestlé Research, CH-1000 Lausanne, Switzerland; 2 Department of Data Sciences and Precision Nutrition, Nestlé Institute of Health Sciences, Nestlé Research, CH-1000 Lausanne, Switzerland; 3 Department of Bioanalytics, Nestlé Institute of Food Safety and Analytical Sciences, Nestlé Research, CH-1000 Lausanne, Switzerland

**Keywords:** *Faecalibacterium prausnitzii*, modeling, inositol, butyrate, American Gut Project, in vitro fermentation

## Abstract

*Faecalibacterium prausnitzii* (*F. prausnitzii*) is a bacterial taxon in the human gut with anti-inflammatory properties, and this may contribute to the beneficial effects of healthy eating habits. However, little is known about the nutrients that enhance the growth of *F. prausnitzii* other than simple sugars and fibers. Here, we combined dietary and microbiome data from the American Gut Project (AGP) to identify nutrients that may be linked to the relative abundance of *F. prausnitzii*. Using a machine learning approach in combination with univariate analyses, we identified that sugar alcohols, carbocyclic sugar, and vitamins may contribute to *F. prausnitzii* growth. We next explored the effects of these nutrients on the growth of two *F. prausnitzii* strains in vitro and observed robust and strain-dependent growth patterns on sorbitol and inositol, respectively. In the context of a complex community using in vitro fermentation, neither inositol alone nor in combinations with vitamin B exerted a significant growth-promoting effect on *F. prausnitzii,* partly due to high variability among the fecal microbiota community from four healthy donors. However, the fecal communities that showed an increase in *F. prausnitzii* on inulin also responded with at least 60% more *F. prausnitzii* on any of inositol containing media than control. Future nutritional studies aiming to increase the relative abundance of *F. prausnitzii* should explore a personalized approach accounting for strain-level genetic variations and community-level microbiome composition.

## 1. Introduction

*Faecalibacterium prausnitzii* (*F. prausnitzii*) belongs to the *Ruminococcaceae* family (phylum *Firmicutes*) and is one of the most abundant bacteria in the human gut [1]. It has been demonstrated to be associated with (the severity or incidence) of different diseases in humans and to play a causative role in mouse models [1]. Reduced abundance of *F. prausnitzii* has consistently been found in disease conditions such as inflammatory bowel disease (IBD) [2], irritable bowel syndrome (IBS), metabolic syndrome and diabetes [3,4,5,6], non-alcoholic fatty liver disease (NAFLD) and non-alcoholic steatohepatitis (NASH) [7], colorectal cancer (CRC) [8], obesity, and frailty [9]. Functionally, *F. prausnitzii* contributes to the modulation of the immune system and attenuation of inflammation through multiple mechanisms that can work independently or synergistically. More precisely, butyrate produced by *F. prausnitzii* and other butyrate-producing bacteria reduces intestinal mucosal inflammation by inhibiting nuclear factor kappa-light-chain-enhancer of activated β-cells (NF-κβ) activation, upregulating peroxisome proliferator-activated receptor-γ expression, and inhibiting interferon-γ expression [9]. In addition, *F. prausnitzii* modulates inflammatory signals by releasing immune-suppressing molecules such as salicylic acid [10] and microbial anti-inflammatory molecules (MAM) [11]. The therapeutic potential of *F. prausnitzii* through the secretion of microbial anti-inflammatory molecules has been demonstrated in a mouse model of IBD [12]. Together with association-based evidence from observational and clinical studies, scientists have argued for the use of *F. prausnitzii* as a probiotic [9].

According to International Scientific Association for Probiotics and Prebiotics (ISAPP), the definition of probiotics is “live microorganisms that, when administered in adequate amounts, confer a health benefit on the host” [13]. *F. prausnitzii* is currently not accepted as a probiotic due to the lack of clinical evidence on its safety and efficacy. The extreme oxygen sensitivity of *F. prausnitzii* imposes practical challenges to the production, transportation, storage, and manufacturing of probiotic products to be evaluated in a clinical setting. Alternatively, the relative abundance of *F. prausnitzii* in the human gut can be affected by multiple factors such as antibiotic usage [14] and diet [15,16]. More precisely, some food ingredients have been shown to increase the abundance of *F. prausnitzii* in humans. Thus, a prebiotic approach aiming to enhance health by increasing the abundance of commensal *F. prausnitzii* could be a viable strategy.

Indeed, *F. prausnitzii*’s relative abundance in the human gut appears to be associated with diet healthiness (based on a healthy eating index) [17]. More specifically, consumption of prebiotic-type ingredients such as inulin and fructo-oligosaccharides was found to increase *F. prausnitzii* in obese women [18], IBS patients [19], and healthy individuals [20]. Treatment with polydextrose and chickpea oligosaccharides (raffinose) also leads to the increase in *F. prausnitzii* abundance in fecal communities of healthy subjects [21,22]. Yet, deconvoluting the effects of individual nutrients or food items on *F. prausnitzii* in the gut from the rest of the diet remains challenging.

Thus, our aim was to identify nutrients that could be used to boost *F. prausnitzii* abundance in the human gut. To this end, we applied a machine learning algorithm on dietary records and 16S rRNA gene sequencing data collected on 3816 participants of the American Gut Project (AGP) to identify new nutrients that may link to the relative abundance of *F. prausnitzii*. We next evaluated the impact of selected nutrients on the growth of *F. prausnitzii* in vitro using pure culture of single strains and fermentation of healthy human fecal communities.

## 2. Materials and Methods

### 2.1. Data

The intersection of three datasets (metadata, microbiota, and VioScreen food frequency questionnaires (FFQ)) from American Gut Project (AGP) [23] was used in this study and represented a sample size of *n* = 3816 (Supplementary Figure S1). Raw 16S rRNA gene sequencing data from stool samples was downloaded from the Qiita repository https://qiita.ucsd.edu/study/description/10317 (accessed on 4 September 2019) [24]. Data were processed following the same analytical steps as in the original publication [23]. Briefly, raw sequencing reads were firstly denoised and sub-operational taxonomic units (sOTUs) were generated using deblur v. 1.0.2 [25]. Then, sOTUs matching bacteria potentially blooming under room temperature storage conditions were removed following the instructions of https://github.com/knightlab-analyses/bloom-analyses (accessed on 31 July 2019). Multiple rarefactions were performed 10 times at a threshold of 1250 sequences per sample. Finally, representative sequences of each sOTU were annotated using the QIIME2 v. 2017.4 RDP classifier on Greengenes 99% v. 13.8 [26]. Nutrient data as provided through VioScreen FFQ analysis were downloaded from the AGP data File Transfer Protocol (FTP) site http://ftp.microbio.me/AmericanGut/raw-vioscreen/vioscreen_dump.tsv.gz (accessed on 31 July 2019). Coded names and full descriptions of the nutrients and the corresponding units of nutrients are shown in Supplementary Table S1. The metadata file “10317_20220801-114642.txt” was downloaded on from the Qiita repository https://qiita.ucsd.edu/study/description/10317 (accessed on 15 September 2022).

### 2.2. Modeling to Predict the Abundance of F. prausnitzii Using Nutrient Intake Data

Predictive models were built to determine the relative abundance of *F. prausnitzii* of an individual subject based on nutrient intake values. In particular, the model predicted the *F. prausnitzii* relative abundance by several nutrient feature parameters to determine the *F. prausnitzii* abundance category of the subject as defined in Supplementary Table S2 (e.g., “Low” or “notLow”; “High” or “notHigh”; “Low” or “High”). A cube root transformation was applied to the *F. prausnitzii* abundances to make them normally distributed before binning them into these different categories (Supplementary Figure S2).

Data were split into a training set “Train” (80%) and a testing set “holdout/Test set” (20%). For optimal model performance, we used random downsampling to match the number of subjects between the abundance groups. For example, we randomly downsampled in notLow group to match the subject number of Low group. The Train set was used by different machine learning algorithms (RandomForests, XGBoost; available from scikit-learn in Python programming language [27]) to train a model. The learning from the data was completed in a cross-validated manner where Train data were split into partitions with some parts used for training the model and others for internal testing (repeated k-fold cross-validation, i.e., 3 folds, 3 repeats). The holdout/Test set was used only for checking the performance of the final trained model and was not used during the model training phase. A total of 9 models (model A to model I) were made, and the cut-offs used to define the groups, the type of machine learning algorithm used, and other parameters for each of the models are provided in Supplementary Table S2.

Receiver operating characteristic (ROC) curves were generated for these models and area under the curve (AUC) was reported. The best performing model was then selected from the different binning categories of Low vs. notLow, High vs. notHigh, and Low vs. High (Supplementary Table S2).

### 2.3. Culture Conditions for Testing Selected Nutrients 

*F. prausnitzii* strains A2-165 and 27768 were obtained from the Deutsche Sammlung von Mikroorganismen und Zellkulturen GmbH (DSMZ, Leibniz Institute, DSMZ German Collection of Microorganisms and Cell Cultures, Braunschweig, Germany) and American Type Culture Collection (ATCC), respectively. Culture of *F. prausnitzii* followed the method of Duncan et al. [28] using Hungate culture tubes in an anaerobic chamber (H_2_:CO_2_:N_2_, 5:10:85%, Type B, Coy Laboratory Products, Grass Lake, MI, USA). To prepare the working cultures, lyophilized *F. prausnitzii* (ATCC 27768) were enumerated anaerobically with 20 mL ATCC media 2107 consisting of trypose 10 g/L, beef extract 10 g/L, yeast extract 3 g/L, dextrose 5 g/L, NaCl 5 g/L, starch 1 g/L, L-cysteine HCl 0.5 g/L, sodium acetate 3 g/L, resazuim (0.025%) 4 mL/L in dd water for 3 days. For each experiment, 0.5 mL of homogenized live liquid culture was added to 9 mL freshly prepared yeast casitone fatty acid broth (YCFA) media in a Hungate tube under an anaerobic condition. Growth of bacteria was evaluated by the measurement of optical density at 600 nm (Biowave WPA CO8000—WPA Cambridge, UK) after incubation at 37 °C on a rotating platform inside of an anaerobic chamber. 

Glucose (Sigma-Aldrich, Schaffhausen, Switzerland) at 10 mM was used as a control carbohydrate source to verify that the strains grew under the assay conditions. To test the ability of *F. prausnitzii* to grow on different carbon sources, glucose was replaced by the same concentration of inositol, sorbitol, erythritol, pinitol, or xylitol prepared with sterile ddH_2_O that was pre-flushed with N_2_ gas. The effect of vitamins was tested at the final concentration of 1 μg/10 mL for vitamins B5 and B6, at 0.05 μg/10 mL for vitamin B12 or 0.1 μg/10 mL for vitamin A and D in the YCFA media with either glucose or inositol as the main carbon source. YCFA media were autoclaved at 121 °C for 15 min and were transferred to an anaerobic chamber till use. L-cysteine-HCl, thiamine hydrochloride (T4625-5G, Sigma-Aldrich, Schaffhausen, Switzerland), and riboflavin (R4500, Sigma-Aldrich, Schaffhausen, Switzerland) were first sterile filtered (0.2 μm, media bottle filtration unit with polyethersulfone (PES) membrane, VWR No. 514-0297) and added to the media prior to each experiment and the pH was adjusted to 6.7 with NaOH immediately before the start of the experiment.

### 2.4. Batch Fermentation

Fecal samples of healthy volunteers were collected under a protocol approved by Lausanne ethical committee (CER-VD) (authorization number: 2020-00304). Inclusion criteria are healthy participants aged 18–60 years old who provide informed consent and are willing to follow the clinical study protocol. The exclusion criteria are (1) following a particular dietary regime such as vegan, vegetarian, ketogenic, or paleo diet; (2) experiencing chronic or recurrent diarrhea with spontaneous bowel movement more than twice a day; (3) antibacterial/antifungal therapy during the 3 months prior to study enrollment; (4) medications or supplements that are known to alter gut function or gut microbiota (i.e., acid antisecretory drugs, pre-/probiotics supplements, laxatives) during the 4 weeks prior to study enrolment, (5) prior gastrointestinal surgery, (6) alcohol intake higher than 2 servings per day; (7) artificially sweetened beverage intake higher than 1000 mL/per day; (8) current or history of gastrointestinal diseases. Preparation of stool samples for in vitro fermentation followed the procedure described by Van den Abbeele et al. [29]. Freshly collected stool samples were placed in an air-tight jar equipped with AnaeroGenTM (Sigma-Aldrich, Schaffhausen, Switzerland) to reduce exposure to ambient oxygen. Once inside an anaerobic chamber (Coy Laboratory Products, Grass Lake, MI, USA), fecal materials were diluted 10 times (*w*/*v*) in anaerobic phosphate buffer (0.1 M of NaH_2_PO4 and 0.1 M of Na_2_HPO4 in 2:1 ratio) containing 10% glycerol and the aliquots of fecal stocks (25 mL) were stored at −80 °C for later use. In vitro fermentation experiment carried in Hungate tubes where 0.25 mL of fecal stock solution was inoculated to 10 mL of a casitone-supplemented oligotrophic medium: casitone (10 g/L), L-cysteine (0.05%), NaCl (8 g/L), KCl (0.2 g/L), Na_2_HPO_4_ (1.15 g/L), KH2PO4 (0.2 g/L) at pH 7.3 as starting of fermentation [30]. A total of four different fecal samples were tested in this study. Inulin or inositol at 10 mM was added to the basic culture media, and vitamins B5, B6, B12, A, and D were included in the relevant groups at the same concentrations as pure culture experiments mentioned above. Samples were collected at times 0, 6 h, 24 h, and 48 h from the start of the experiment for the quantification of *F. prausnitzii* and metabolomic analysis.

### 2.5. Bacterial DNA Extraction

Bacterial DNA was extracted using QIAamp Fast-DNA Mini Kit (Qiagen, no: 51604, Hilden, Germany) following the manufacturer’s recommended procedure. In short, in vitro fermentation samples (1 mL) were mixed an equal amount of InhibitEX buffer (Qiagen, Hilden, Germany) in a Lysing Matrix B tube before two steps of homogenization with Fastprep (M.P. Biomedicals, Irvine, CA, USA). Lysate was further prepared by centrifugation, proteolytic digestion with Protease K, and incubation (10 min). Then, DNA was extracted and purified with QIAamp spin column. The concentration of resulting DNA was measured by fluorescent method using Varioskan Lux (ThermoFisher Scientific, Zug, Switzerland) and PicoGreen (ThermoFisher Scientific, Zug, Switzerland). DNA samples were stored at −20 °C before the quantification of *F. prausnitzii*.

### 2.6. Quantification of Total Bacteria and F. prausnitzii by Real Time PCR

Quantification of total bacteria and *F. prausnitzii* was performed with real-time PCR using the ABI-PRISM 7700 Sequence Detection System (Applied Biosystems, Foster City, CA, USA) in duplicates. Quantification of total bacteria was performed in a total volume of 25 µL reagent mix using the Perfecta MasterMix (Quantabio, PerfeCta^®^ qPCR ToughMix^®^ ROX, Beverley MA, USA), containing 300 nM of each of the forward (f: TCCTACGGGAGG CAGCAGT) and reverse primers (r: GGACTACCAGGG TATCTAATCCTGTT) and 175 nM of fluorogenic probe (FAM-CGTATTACCGCG GCTGCTGGCAC-BHQ) as described by Nadkarni et al. [31]. The amplifications of DNA were 95 °C for 10 min and 50 cycles of 95 °C for 15 s and 60 °C for 1 min. Detection of *F. prausnitzii* follows the method described by Lopez-Siles et al. [32]. In short, PCR reactions were carried out in 20 µL containing TaqMan Universal PCR Master Mix, 300 nM of each of the forward (Fpra 428 F TGTAAACTCCTGTTGTTGAGGAAGATAA) and reverse (Fpra 583 R GCGCTCCCTTTACACCCA) primers and 200 nM of Probe (Fpra 493 PR 6FAM-CAAGGAAGTGACGGCTAACTACGTGCCAG-TAMRA). Data analysis made use of Sequence Detection Software version 1.6.3 supplied by Applied Biosystems (Foster City, CA, USA).

### 2.7. ^1^H-Nuclear Magnetic Resonance (NMR) Metabolomics

Frozen samples from in vitro fermentation were thawed at room temperature before centrifugation for 10 min at 10,000× *g* at 4 °C. The supernatants (300 μL) were added to 300 μL sodium phosphate buffer 0.075 M at pH 7.4, vortex mixed and 560 μL were transferred to 5 mm NMR tubes. The samples were then analyzed by 1D ^1^H-NMR in a 600 MHz Bruker spectrometer at 300 K. A set of 2D NMR experiments (^1^H J-Resolved, ^1^H-^1^H COSY, and ^1^H-^13^C HSQC) were acquired for selected samples to aid metabolite identification. All NMR spectral acquisition and pre-processing were completed under the control of TopSpin 4.0.9 (Bruker BioSpin, Rheinstetten, Germany), and the automated submission of a sequence of samples was performed using ICON-NMR 5 (Bruker BioSpin, Rheinstetten, Germany). Metabolite annotation was performed by comparing metabolite signals to those of Bruker BIOREFCODE library and public database Human Metabolome Database (HMDB) [33].

To analyze the data, 1D NMR spectra were imported into R statistical software environment (version 4.1.1, R Foundation for Statistical Computing, Vienna, Austria) [34] using the AlpsNMR package [35], and intensities and chemical shifts were interpolated to obtain a consistently shared ppm axis for all spectra between −0.5 and 10 ppm. Residual signal of water (4.70 to 4.9 ppm) was removed. Targeted peak integration was performed using a numeric integration automated routine in R statistical software. The integrated data were log-transformed prior to statistical analysis. Metabolic profile was visualized by a principal component analysis (PCA) performed using unit-variance scaling.

### 2.8. General Statistical Analysis

Comparisons of *F. prausnitzii* relative abundance between groups were performed with Kruskal–Wallis rank sum test followed by post hoc Dunn test. Wilcoxon rank-sum test was used to compare the intake of nutrients between Low and notLow *F. prausnitzii* categories and Benjamini–Hochberg method was applied to control the false discovery rate (0.05). Descriptive statistics on the Healthy Eating Index-2010 were based on data published by the United States Department of Agriculture (https://fns-prod.azureedge.us/sites/default/files/media/file/HEI2010_Age_Groups_2011_2012.pdf, accessed on 1 December 2022). The above analyses were performed using the R statistical software, v 4.1.1., R Foundation fo Statistical Computing, Vienna, Austria.

Data are expressed as mean ± Standard error of the mean (SEM) for data from in vitro culture and fermentation experiments. Comparisons between the groups were examined with one-way ANOVA followed by Tukey multiple comparisons test using GraphPad Prism version 9.2.0 for Windows (GraphPad Software, San Diego, CA, USA).

## 3. Results

### 3.1. Characteristics of the Study Subjects

We used microbiome and dietary data collected on 3816 individuals from the AGP cohort [23]. The full description of the cohort is shown in Supplementary Table S3. There is a higher percentage of females (59.4%) than males (38.9%), and participants’ self-reported country of residence is primarily from the US (43.3%), followed by the UK (21.7%) and Australia (1.5%). The average age of the study population is 51.3 ± 15.6 years old (mean ± standard deviation (SD)), and a large portion falls into the normal Body Mass Index (BMI) category (54.9%) with some being overweight (28.9%), obese (9.5%) and underweight (4.4%). In terms of dietary preference, 76.8% declared as omnivores and the remaining subjects follow vegetarian (4.8%), vegan (3.2%), and other, e.g., tribal diets (Supplementary Table S3). Quality of nutrition intake as measured by Health Eating Index (HEI) is 66.34 ± 1.38 for children (2–17 years, *n* = 68), 70.8 for adults (18–64 years, *n* = 2686), and 71.54 ± 0.32 for older adults (≥65 years, *n* = 853). The HEI scores appear to be higher in all age groups of AGP subjects than in the age-matched general US population (NHANES 2011–2012), although a statistical comparison was not possible due to the different methods in collecting dietary intake information (Table 1). Interestingly, we found significant declines in *F. prausnitzii* abundance with age (Kruskal–Wallis rank sum test, *p* = 6.9 × 10 − 5, Dunn test 20 s vs. 50 s adj *p* = 0.04; 20 s vs. 60 s adj *p* = 0.03, Supplementary Figure S3).

### 3.2. Discovery of Nutrients Associated with the Abundance of F. prausnitzii

**Figure 1:**
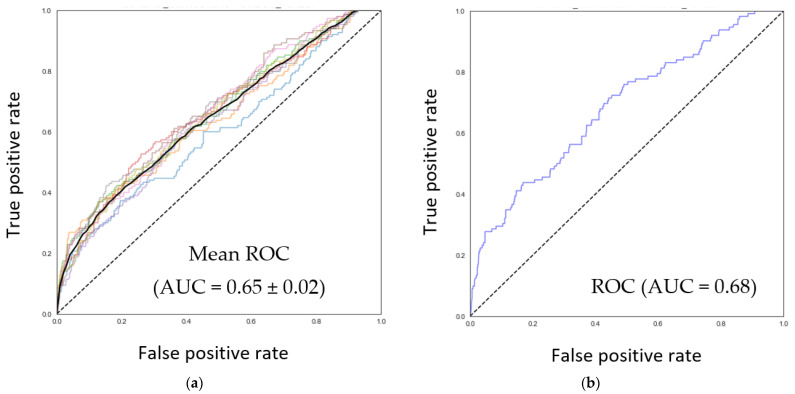

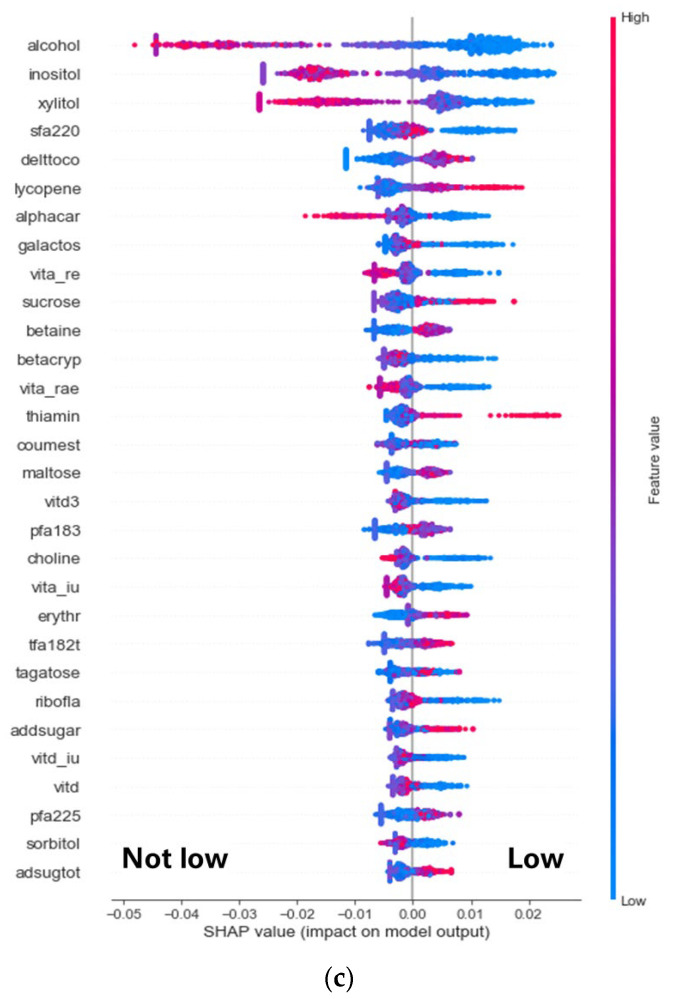
Prediction of relative abundance of *F. prausnitzii* from nutrient intake data. A random forest model was used to predict the individual’s relative abundance of *F. prausnitzii* in low or notlow category (<mean—1SD vs. the rest). ROC curves and area under the curve (AUC) values for the training (in cross validation mode with 3 folds, 3 repeats) (**a**) and test (**b**) datasets are shown. Different colors in (**a**) indicate performance under different model runs (3 folds, 3 repeats) and the black line indicates an averaged out Mean performance. Top 30 most important nutrient features of the model are shown in (**c**), where contributions of each nutrient feature to the model was analyzed by SHAP. Each dot represents an individual sample. Red and blue color gradations depict higher and lower value of the nutrient’s intake, respectively. If a feature has red values towards the right of the vertical line at 0.00, this indicates higher values of this feature contribute towards “Low” model output. Vice versa, if a feature has red values towards the left of the vertical line at 0.00, this indicates higher values of this feature contribute towards “notLow” model output.

To identify nutrients that can predict the relative abundance of *F. prausnitzii* in the gut ecosystem as estimated from fecal sampling, random forest and XGBoost machine learning models with three-fold cross-validation were generated using 251 nutrition-related features extracted from FFQs. Among all considered models, model E (Low vs. notLow with cut-off based on mean—1SD, Supplementary Table S2) performed the best with an AUC-ROC of 0.65 ± 0.02 for the training (*n* = 896) and 0.68 (*n* = 764) for the test set (Figure 1a,b).

Using the agnostic technique SHapley Additive exPlanations (SHAP) [36] to explain predictions of the model, we identified positive contributions of inositol, xylitol, saturated fatty acid 22:0, a-carotene, galactose, and vitamin A to the abundance of *F. prausnitzii* whereas d-tocopherol, lycopene, sucrose, and betaine displayed a negative relationship (Supplementary Figure S4A–L).

To complement the above results, we performed univariate analysis (Kruskal–Wallis test) to compare nutrient intakes between the population split according to *F. prausnitzii* relative abundance being Low or notLow, using the same definition of the bins as the best abovementioned model. A total of 11 nutrients were significant after passing the false discovery rate (Wilcoxon rank sum test, adjusted p-value (*p*.adj) < 0.05, e.g., alcohol, inositol, aspartame, beta-cryptoxanthin (betacryp), beta-carotene (betacar), total vitamin A activity International Units (vita_iu), total vitamin A activity retinol equivalents (vita_re), alpha-carotene (alphacar), pectins, total vitamin A activity retinol activity equivalents (vita-rae) and lutein + zeaxanthin (lutzeax) (Supplementary Table S3)). When comparing the two *F. prausnitzii* groups (low and notlow) with nutrient intakes (Supplementary Table S4) or intake normalized to 2000 kcal, alcohol, inositol, aspartame, betacryp, and alphcar remained significantly different (Supplementary Table S5, Wilcoxon rank sum test, *p*.adj < 0.05).

### 3.3. Growth of F. prausnitzii on Inositol-Based Media Is Strain Dependent

**Figure 2:**
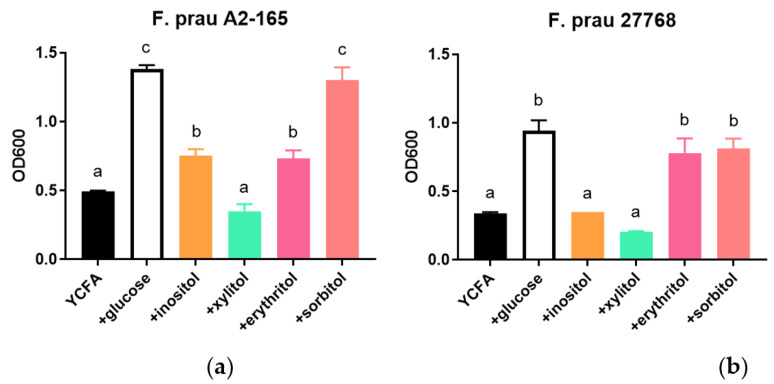
Growth promoting effects of sugar alcohols in two strains of *F. prausnitzii*. Experiments were performed with DSMZ A2-165 (**a**) or ATCC 27768 (**b**) in a YCFA-based media supplemented with different carbon substrates for 48 h in an anaerobic condition. Results are mean ± SEM, *n* = 3. Statistical analysis was performed with ANOVA followed by Tukey post hoc analysis. When treatment groups share a same letter, it means no statistical difference between the groups.

Out of the top nutrients featured in the above analyses, few have previously been shown to support the growth of *F. prausnitzii* in a culture condition, namely: sucrose, maltose, and galactose [28,37]. We, therefore, selected some of those nutrients to test their potential to enhance *F. prausnitzii* growth in vitro, namely: carbocyclic sugar (i.e., inositol) and sugar alcohols (i.e., xylitol, and sorbitol; Figure 1c and Supplementary Figure S4A,B,K). Briefly, we measured the growth of two strains of *F. prausnitzii* 27786 and A2-165 representing different phylogenic groups of the bacteria [38] for a period of 48 to 72h on either inositol, xylitol, erythritol, or sorbitol as a primary carbon source in a YCFA media. We observed that growth under the various tested conditions was strain dependent. Growth of *F. prausnitzii* A2-165 on media prepared with sorbitol was comparable to that observed with glucose as the most efficient carbon substrate followed by inositol and erythritol (Figure 2a) and was further diminished with xylitol to a level close to that with basic YCFA media without any carbon substrate (*p* = 0.0581). In contrast, the ATCC 27768 strain grew equally on glucose, erythritol, and sorbitol equally, while inositol and xylitol failed to support its growth (Figure 2b).

**Figure 3:**
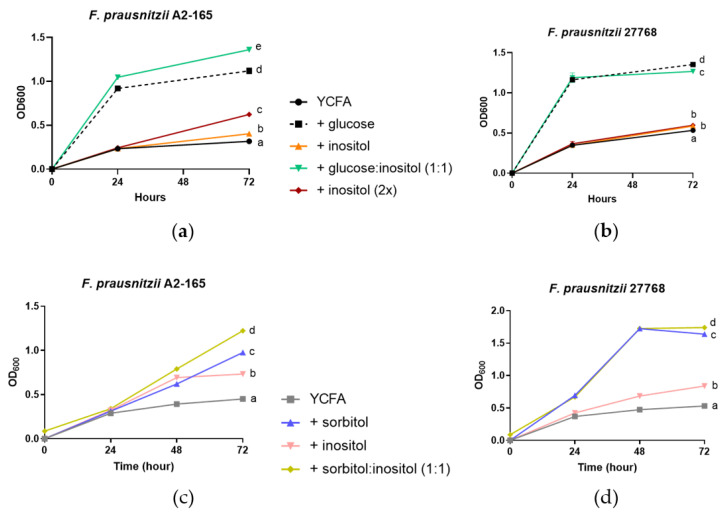
Strain-dependent growth response to a combination of nutrients. Growth of *F. prausnitzii* in culture with single nutrient or in combination was followed for 72 h and optical density was measured every 24 h. Glucose (G), inositol (I), glucose and inositol (G + I), and twice the amount of inositol (2I) were added to a YCFA media and the results of growth for DSMZ A2-165 and ATCC 27768 are reported in (**a**,**b**), respectively. Sorbitol (S), inositol (I), or a combination of sorbitol and inositol at equal amounts (1:1) were tested in DSMZ A2-165 (**c**) and ATCC 27768 (**d**) strains for 72 h. Results are mean ± SEM, *n* = 3. Statistical analysis of 72 h data was performed with ANOVA followed by Tukey post hoc analysis. When treatment groups share a same letter, it means no statistical difference between the groups.

We next investigated whether *F. prausnitzii* responds differently with increasing amounts of inositol or in combination with other carbon sources. On the inositol-based YCFA media, the growth of A2-165 and 27768 strains only marginally increased compared with YCFA alone (Figure 3a,b, *p* = 0.0006 for A2-165 and *p* = 0.0004 for 27768). Doubling the amount of inositol in the media led to 55% more growth with the A2-165 strain (Figure 3a, *p* < 0.0001) but not with the 27768 strain (Figure 3b, *p* = 0.7151) when compared with normal amounts of inositol. To further illustrate the strain-specific substrate utilization, a combination of glucose with inositol also increased the growth of strain A2-165 by 21.4% (*p* < 0.0001) compared to glucose alone, while the combination slightly reduced the growth of 27768 by 6.3% (*p* < 0.0001). Finally, the addition of inositol to sorbitol promoted the growth of the A2-165 strain by 64.4% and 23.7% compared to sorbitol alone (*p* < 0.0001), while only a minimal effect of 6.1% was observed on strain 27768 (*p* < 0.0001; Figure 3c,d).

To further support the predictive potential of the machine learning approach, we also tested whether nutrients predicted to have a negative impact on *F. prausnitzii* would have similar effects experimentally. Results showed that lycopene significantly suppressed the growth of A2-165 by 31.4% (*p* = 0.039), especially with glucose as the main carbon source (Supplementary Figure S5A) while betaine failed to alter the growth pattern of the A2-165 strain (Supplementary Figure S5B).

### 3.4. Responses of F. prausnitzii to Nutrients in a Mixed Community

In a mixed community such as the human gut microbiota, *F. prausnitzii* may compete or work synergistically with other species for nutrients. Hence, the response of *F. prausnitzii* to nutrients may highly depend on an ecological context, which could explain the discrepancy between the model predictions and the in vitro observations described above. Therefore, we tested the effects of nutrient supplementation on *F. prausnitzii* growth by quantitative PCR (qPCR) in an in vitro fermentation system with adult human stool samples. Inositol was chosen as the main energy source instead of sorbitol because sorbitol has not been shown to affect the composition of gut microbiota [39] and inositol consistently differentiated *F. prausnitzii* categories in machine learning and univariate analyses. In addition, studying isolated sorbitol outsides of fruits and vegetables limits the translational value because sorbitol as a part of Fermentable Oligosaccharides, Disaccharides, Monosaccharides, and Polyols (FODMAP) is not well tolerated by some people [40]. Inositol was also tested alone (inositol) or in combination with B vitamins, specifically B5, B6, and B12 (VitBs + inositol). A dedicated group with only the three vitamin Bs without inositol (VitBs) was established because they were not only predicted by the model but also essential for *F. prausnitzii* [41]. Moreover, we included vitamins A and E which were also identified in the models together with inositol, and B vitamins (B5, B6, and B12) to create a comprehensive nutrition mixture (Full). Finally, inulin was used as positive control based on previous reports of a positive effect on the growth of *F. prausnitzii* [39].

**Figure 4:**
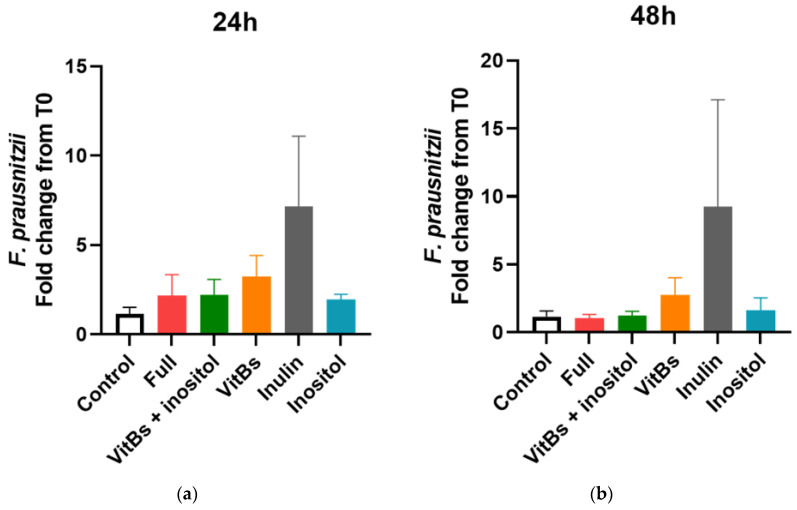

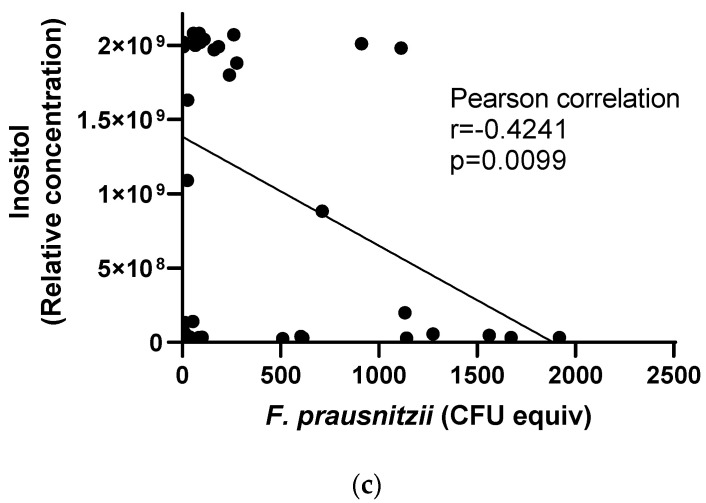
Evaluation of nutrients and nutrient combinations on the growth of *F. prausnitzii* in a mixed community. Details of in vitro fermentation experiments are described in Methods and Materials. The fold changes in *F. prausnitzii* at 24 h (**a**) and 48 h (**b**) from the baseline were calculated from results of *F. prausnitzii-*specific qPCR quantification. Each treatment was tested in 4 fecal samples and data are shown in mean ± SEM. Statistical analysis was performed with ANOVA followed by Tukey post hoc analysis, but no statistically significant difference was found. Relationship between inositol and the amount of *F. prausnitzii* is shown in (**c**). Signals of butyrate were extracted from ^1^H-NMR metabolomics as expressed as relative concentration and the amount of *F. prausnitzii* was determined with qPCR technique shown as CFU equivalent (equiv). Data were pooled from the groups with added inositol at 6, 24, and 48 h time points. Each (•) corresponds to a sample taken from the in vitro fermentation experiment. Pearson correlation analysis indicates a positive relationship between the number of *F. prausnitzii* and butyrate (*p* = 0.0099, *r* = 0.4241).

Effects of nutrients on *F. prausnitzii* growth were tested for a period of 48 h using fecal samples from four individual donors as replicates in a casitone-based oligotrophic media. With most interventions, we observed a non-significant increase in *F. prausnitzii* compared to the control, especially after 24 h (Figure 4a,b). A high degree of heterogeneity in the response was observed across fecal donors (Supplementary Figure S6A–D). For instance, treatment with inulin resulted in a 24.5- and 10.6-fold increase in *F. prausnitzii* at 24 h compared to control in donor 2 (D2) and 3 (D3), respectively, while no effects were observed with donor 1 (D1) and 4 (D4). Inositol alone or inositol with vitamin supplementations also triggered an increase in *F. prausnitzii* by at least 50% compared to control in D2 and D3 communities, and yet no effects were observed with D1 and D4 (Supplementary Figure S6A–D). Next, we performed regression analysis to examine the relationship between inositol and *F. prausnitzii*. Only data from the groups with added inositol and the time points 6, 24, and 48 h were included in the analysis. As shown in Figure 4c, the number of *F. prausnitzii* is weakly and inversely associated with the amount of inositol, a result in line with the single strain experiments mentioned above.

**Figure 5:**
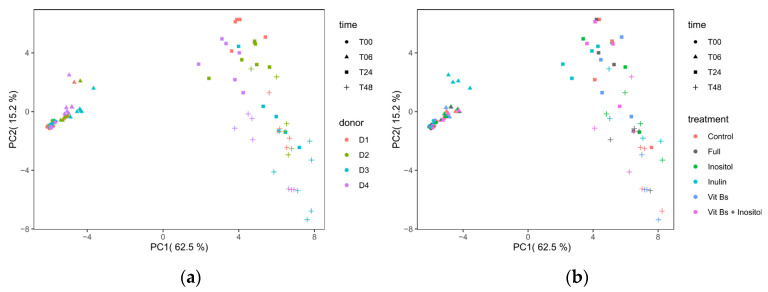

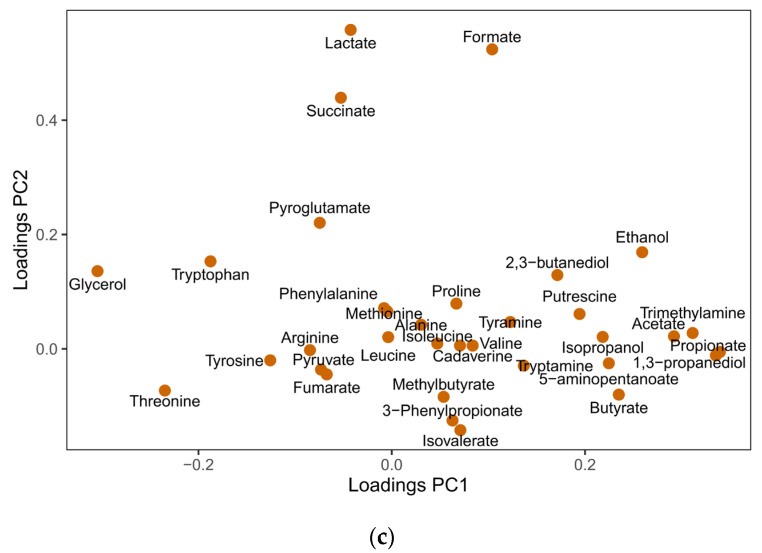
PCA analysis of metabolomic profiles of in vitro fermentation media. Effects of nutrient or nutrient combinations were examined in four microbiota backgrounds (D1, D2, D3 and D4 corresponds to donor 1, 2, 3 and 4). Samples were collected at times 0, 6, 24, and 48h (T00, T06, T24 and T48) from batch fermentation experiments. Each dot in the PCA score plot represents an individual, and the shape and color of symbol denote the time point, fecal donor, or nutrition treatment (**a**,**b**), respectively. The loading plot highlighted the metabolites influencing distribution in PCA score plots (**c**).

To further understand the heterogeneity of these results, we next conducted metabolomics profiling of the fermented media at all time points. PCA with 34 identified and integrated NMR signals of metabolites suggested that time had more effects on the metabolomic variance during the fermentation than donor or treatment. While the 6 h time points clustered closely with the baseline samples, a drastic change in the overall metabolic profile was observed at 24 and 48 h (Figure 5a,b). PCA loadings showed that from 6 h to 24 h of fermentation, short-chain fatty acids, trimethylamine, alcohols, monamine aromatic amino acid-derivatives, diamines, and related metabolites increased, while glycerol and some amino acids (threonine, tryptophan, tyrosine, and arginine) decreased (Figure 5c). Lactate, formate, and succinate increased over 24 h before being consumed at 48 h.

**Figure 6:**
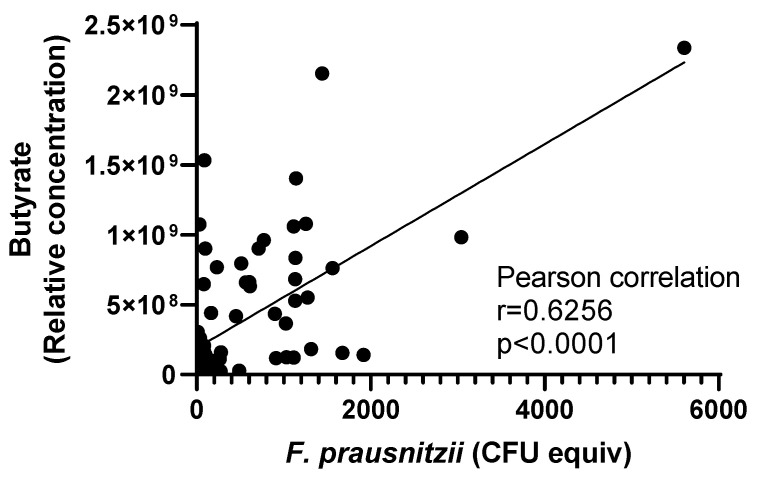
Relationship between butyrate and the amount of *F. prausnitzii* in batch fermentation experiments. Signals of butyrate were extracted from ^1^HNMR metabolomics as expressed as relative concentration and the amount of *F. prausnitzii* was determined with qPCR technique shown as CFU equivalent (equiv). Data were pooled from 6, 24, and 48 h time points with all treatment groups in all four donors. Each (•) corresponds to a sample taken from the in vitro fermentation experiment. Pearson correlation analysis indicates a positive relationship between the number of *F. prausnitzii* and butyrate (*p* < 0.0001, *r* = 0.6256).

Focusing more precisely on the metabolization of the tested substrates, we observed in all four donors, that inositol was fully consumed over time (Supplementary Figure S7A–C) independently of vitamin supplementation, and the rate of consumption was not related to the level of *F. prausnitzii*. Finally, as *F. prausnitzii* is one of the key butyrate-producing bacteria [42], we examined the correlation between *F. prausnitzii* and butyrate in the batch fermenters. Butyrate levels were undetectable at times 0 and 6 h and were indistinguishable among groups at 24 h (Supplementary Figure S8A). At 48 h, butyrate was higher in the inulin group than in the Full and VitBs+inositol groups (Supplementary Figure S8B). Taking all samples into account, we observed a positive correlation between levels of *F. prausnitzii* and butyrate concentration (Pearson correlation, *r* = 0.6252, *p* < 0.0001; Figure 6). Even after removing the leverage point, the result is still significant (*p* < 0.0001, *r* = 0.4661). This correlation remained significant when considering D3 alone while only a trend was observed for D1 and D4 (Supplementary Figure S9).

## 4. Discussion

*F prausnitzii* is amongst the most abundant anaerobic bacteria in the human gut, and scientific evidence supports its beneficial role in health. In the present study, we applied a machine learning algorithm to microbiome composition and food frequency questionnaires data collected on 3816 AGP participants and identified nutrients that may influence the abundance of *F. praustnizii*. Many of the top nutrients, such as galactose (rank #8), sucrose (rank #10), and maltose (rank #16) have been shown in the literature to support the growth of *F. prausnitzii* in a culture condition. We, therefore, focused on examining the potential effect of other nutrients on the growth of *F. prausnitzii* in vitro. Subsequent in vitro experiments with two strains of *F. prausntizii* demonstrated that inositol, sorbitol, and lycopene could enhance the growth of at least one of the selected bacterial strains as predicted by the model. On the contrary, xylitol, erythritol, and betaine failed to increase *F. prausnitzii* growth under in vitro conditions suggesting that other factors than these nutrients alone may be at play. More importantly, we observed strain-dependent responses of *F. prausnitzii* to most nutrients or nutrient combinations. In addition, when the effects of nutrients on *F. prausnitzii* were tested in the context of complex communities using in vitro fermentation, we observed a high degree of variations among the four fecal donors, rendering no significant changes in the number of *F. prausnitzii*. Interestingly, we observed a significant positive correlation between *F. prausnitzii* and butyrate concentration during fermentation, supporting the use of in vitro fermentation models to study microbial metabolism.

A citizen science project such as AGP offers a large dataset for examining the relationships between gut microbiota and a wide variety of factors such as dietary patterns, lifestyle, diseases, etc. [23,43,44,45,46]. We included previously unprocessed 16S composition data and created a cohort of 3816 AGP subjects for this study. Compared to the typical American adult population (NHANES), the study cohort seemed to consume fewer calories and have healthier eating habits for children, adults, and to a lesser extent for older adults. Contrary to calorie intake, the AGP cohort reported a higher intake of fiber and vitamin B12 than NHANES, further supporting a healthy eating choice of AGP participants. It is, however, worthwhile to mention that different methods of collecting dietary intake in the two studies hindered us from performing direct comparisons between the two cohorts, similar to the conclusion of a recent study looking at dietary patterns of 1800 AGP participants [43].

Issues around over-optimism in microbiome analysis have recently been raised. Critiques on overfitting of data point out the potential pitfalls in reliability and reproducibility of the analysis [47]. In the current study, the performance of the model was stable with the AUC value of ROC being slightly higher in the test set than in the training set. Further, when using the same cuts-off to define Low, notLow, different algorithms (i.e., random forest and XGBoost) yielded similar statistical performance, implying that the conclusion was not derived from a selection bias on a particular overfitting model (Supplementary Table S2). Nevertheless, machine learning-based data analysis was meant to generate hypotheses, not conclusions. Through our modeling approach employed here, many nutrients identified in this study were newly associated with *F. prausnitzii*, such as sorbitol and inositol, while others such as alcohol and galactose have been previously reported to positively correlate with high *F. prausnitzii* abundance [32,48]. Inositol or myo-inositol is commonly found in vegetables and meat [49], and sorbitol and many sugar alcohols are found in fruits and vegetables. Evidence from a prospective study and a dietary intervention study showed a positive relationship between the consumption of fruits and vegetables and the abundance of *F. prausnitzii* [50,51], a result in agreement with the findings of our in vitro experiments. On the other hand, we did not observe any growth-promoting effect of xylitol on either strain of *F. prausnitzii*. These results highlight the importance of experimental validations on the outcomes of in silico modeling.

*F. prausnitzii* has a high degree of genetic diversity, and the two strains used in the study (A2-165 and 27768) belong to different phylogroups [37]. The two isolates also showed different growth rates under various dietary and host-derived carbohydrate sources [37]. We also observed strain-specific growth response when inositol was given as a carbon source. Recently, a branch of *F. prausnitzii*, including the A2-165 strain has been reannotated into a new species *Faecalibacterium duncaniae* [52], further highlighting the diverse metabolic potential of *F. prausnitzii*. Interestingly, it was reported that neither *F. duncaniae* nor *F. prausnitzii* grew on inositol, which contrasts with our findings. The discrepancy is likely due to the difference in culture condition: in our study, the growth-promoting effect of inositol was observed after 48 h whereas Sakamoto et al. [52] reported results after 18–24 h incubation time.

Use of in vitro fermentation in a test tube has been widely applied to examine microbial degradation and transformation of prebiotic fibers [53] due to many advantages such as short turnaround time, enhanced throughput, simple equipment setup compared to a continuous system, animal models, or clinical studies [30]. However, it is also the least physiological of all the models as pH is often not fully controlled and waste products are not removed during the fermentation. Another well-known criticism of in vitro systems is the negligence or improper estimation of digestion and absorption of nutrients in the small intestine. Furthermore, interactions between nutrients (e.g., D-glucose and inositol) affect the bioavailability of intestinal tissues [54], thus altering the potential impacts of target nutrients on colonic gut microbiota. Despite the shortcomings of the system, reductionist approaches such as testing nutrients in pure bacteria culture and in complex fecal microbiota provide experimental conditions for testing a causal relationship between nutrients and target bacteria and studying specific functions or metabolites of gut microbiota [55]. In the present study, we showed that inositol was efficiently utilized by all four fecal communities, and *F. prausnitzii* increased at least 1.6-fold over control in three out of four communities. One reason contributing to the interindividual variation could be the differences in microbial composition among all the donor samples. In the present study, we excluded people with conditions such as the use of antibiotics or certain drugs, abnormal bowel movements, GI diseases, and many others that are known to affect the gut microbiota. However, we did not collect dietary intake, lifestyle, ethnicity, and social economic status which are also known factors for causing a shift in microbiota composition. So, the starting microbiota composition in the in vitro experiments might have been less homogenous than we expected, which led to heterogenic responses to the treatments.

*F. prausnitzii* is highly connected with other bacterial members in the energic trophic chain. This is best demonstrated in cross-feeding experiments where the *F. prausnitzii* population benefited from the presence of *Bifidobacteria* and other bacteria for acetate and vitamin Bs, respectively [41,56]. Since *Bifidobacteria* are primary utilizers of inulin in the adult gut ecosystem [57,58], it is not a surprise to see donor-specific responses to test nutrients and inulin. *F. prausnitzii* also compete with other bacteria for carbon sources. As shown by Lopez-Siles et al., *F. prausnitzii* out-competed *Eubacterium eligens* and *Bacteroides thetaiotaomicron* in co-culture experiments with apple pectin. However, it is possible *F. prausnitzii* does not have a competitive advantage over other nutrients. To concretely evaluate the effect of *F. praustnitzii* targeting nutrients, intervention trials in humans coupled with metagenomic and metabolomic analysis are needed to reveal nutrient–*F. prausnitzii* relationship in a complex gut ecosystem.

## 5. Conclusions

In conclusion, we discovered the novel *F. prausnitzii* modulating nutrients using a machine learning approach applied to data from American Gut Project, and many of our predictions were confirmed in in vitro experiments, supporting the value of in silico approach without having a priori hypothesis. Interestingly, sorbitol robustly enhanced the growth of two different strains of *F. prausnitzii* whereas inositol’s effect was strain dependent. While validating the nutrients singly or in combinations, we experienced highly individualized responses among four fecal donors. Although interesting, the results were mainly derived from in silico and in vitro experiments, validation of our findings in humans is required before applying the learnings in this study as a general recommendation or to be considered as a personalized nutrition strategy for enhancing the beneficial gut bacteria such as *F. prausnitzii*.

## 6. Patents

Two patents were filed related to the works discussed here:

(1) Systems and methods for estimating, from food frequency questionnaire-based nutrients intake data, the relative amounts of *Faecalibacterium prausnitzii* (Fprau) in the gut microbiome ecosystem and associated recommendations to improve *Faecalibacterium prausnitzii* [59].
(2) Compositions and methods using at least one inositol or sorbitol to enhance the growth of *Faecalibacterium prausnitzii* [60].

## Figures and Tables

**Table 1:** Comparison * of diet quality measured as healthy eating index between AGP subjects and general American population (NHANES 2011–2012).

Data Source		AGP			NHANES 2011–2012	
HEI-2010 dietary component (max score)	Children 2–17 years (*n* = 68)	Adults 18–64 years (*n* = 2686)	Older adults ≥ 65 years (*n* = 852)	Children 2–17 years (*n* = 2857)	Adults 18–64 years (*n* = 4044)	Older Adults ≥ 65 years (*n* = 1032)
Dairy (10)	5.33 (0.47)	5.03 (0.06)	5.73 (0.09)	9.03 (0.22)	5.78 (0.13)	5.99 (0.16)
EmptyCalories (20)	16.48 (0.49)	17.59 (0.07)	16.91 (0.11)	11.50 (0.28)	12.53 (0.28)	14.99 (0.44)
FattyAcids (10)	5.64 (0.44)	6.01 (0.07)	4.91 (0.12)	3.29 (0.18)	4.92 (0.19)	5.60 (0.36)
GreensAndBeans (5)	3.14 (0.25)	4.39 (0.02)	4.44 (0.04)	0.70 (0.09)	3.63 (0.16)	3.58 (0.47)
RefinedGrains (10)	8.04 (0.37)	9.18 (0.04)	9.46 (0.06)	4.91 (0.16)	6.36 (0.17)	7.34 (0.31)
SeafoodAndPlantProteins (5)	3.26 (0.24)	4.47 (0.02)	4.67 (0.03)	3.05 (0.17)	3.98 (0.22)	4.91 (0.18)
Sodium (10)	3.37 (0.34)	2.43 (0.05)	3.43 (0.09)	4.85 (0.25)	4.04 (0.08)	3.66 (0.26)
TotalFruit (5)	4.38 (0.15)	3.67 (0.03)	4.18 (0.05)	3.91 (0.18)	2.61 (0.11)	3.84 (0.22)
TotalVegetables (5)	3.94 (0.17)	4.69 (0.01)	4.69 (0.03)	2.10 (0.09)	3.54 (0.09)	4.16 (0.19)
WholeGrains (10)	3.93 (0.41)	4.43 (0.07)	3.96 (0.13)	2.50 (0.10)	2.75 (0.16	4.23 (0.34)
TotalScore (100)	66.34 (1.38)	70.73 (0.2)	71.54 (0.32)	55.07 (0.72)	58.27 (0.98)	68.29 (1.76)

* Data are mean (Standard error of the mean). A statistical comparison between the two datasets is not possible. NHANES uses 1–2 24 h recalls, and it is a stratified survey with sampling weights, so that the means and standard errors are weighted. American Gut Project (AGP), on the other hand, is based on food frequency questionnaires (FFQs), and there are no sampling weights.

## Data Availability

Data are available upon request.

## Supplementary Materials

The following supporting information can be downloaded at: https://www.mdpi.com/article/10.3390/nu15061311/s1, Figure S1: Selection of subjects for the analyses; Figure S2: Distribution of *F. prausnitzii* in the AGP sub-cohort; Figure S3: Age-associated relationship with *F. prausnitzii* in AGP sub-cohort; Figure S4: SHAP Dependence plots of top nutrients in the model; Figure S5: The growth of *F. prausnitzii* in single or combination of nutrients; Figure S6: Effect of nutrients on the growth of *F. prausnitzii* in a complex community is donor dependent; Figure S7: PCA analysis of metabolomic profiles of in vitro fermentation media; Figure S8: Decrease in inositol signal over the duration of in vitro fermentation; Figure S9: Relationship between the number of *F. prausnitzii* and butyrate signals in the fermentation media; Table S1: Nutrients in the models; Table S2: Performance of various models; Table S3: Summary of metadata of a subset of American Gut Project participants used in the study; Table S4: Mean intake of nutrients that are significantly different between the Low and notLow *F. prausnitzii* categories; Table S5: Normalized mean intake (2000 kcal) of nutrients that are significantly different between the Low and notLow *F. prausnitzii* categories.

## References

[1] Lopez-Siles M., Duncan S.H., Garcia-Gil L.J., Martinez-Medina M. (2017). Faecalibacterium prausnitzii: From microbiology to diagnostics and prognostics. ISME J..

[2] Zhao H., Xu H., Chen S., He J., Zhou Y., Nie Y. (2021). Systematic review and meta-analysis of the role of Faecalibacterium prausnitzii alteration in inflammatory bowel disease. J. Gastroenterol. Hepatol..

[3] Furet J.P., Kong L.C., Tap J., Poitou C., Basdevant A., Bouillot J.L., Mariat D., Corthier G., Dore J., Henegar C. (2010). Differential adaptation of human gut microbiota to bariatric surgery-induced weight loss: Links with metabolic and low-grade inflammation markers. Diabetes.

[4] Karlsson F.H., Tremaroli V., Nookaew I., Bergström G., Behre C.J., Fagerberg B., Nielsen J., Bäckhed F. (2013). Gut metagenome in European women with normal, impaired and diabetic glucose control. Nature.

[5] Qin J., Li Y., Cai Z., Li S., Zhu J., Zhang F., Liang S., Zhang W., Guan Y., Shen D. (2012). A metagenome-wide association study of gut microbiota in type 2 diabetes. Nature.

[6] Zhang X., Shen D., Fang Z., Jie Z., Qiu X., Zhang C., Chen Y., Ji L. (2013). Human Gut Microbiota Changes Reveal the Progression of Glucose Intolerance. PLoS ONE.

[7] Da Silva H.E., Teterina A., Comelli E.M., Taibi A., Arendt B.M., Fischer S.E., Lou W., Allard J.P. (2018). Nonalcoholic fatty liver disease is associated with dysbiosis independent of body mass index and insulin resistance. Sci. Rep..

[8] Lopez-Siles M., Martinez-Medina M., Surís-Valls R., Aldeguer X., Sabat-Mir M., Duncan S.H., Flint H.J., Garcia-Gil L.J. (2015). Changes in the Abundance of Faecalibacterium prausnitzii Phylogroups I and II in the Intestinal Mucosa of Inflammatory Bowel Disease and Patients with Colorectal Cancer. Inflamm. Bowel Dis..

[9] Martín R., Bermúdez-Humarán L.G., Langella P. (2018). Searching for the Bacterial Effector: The Example of the Multi-Skilled Commensal Bacterium Faecalibacterium prausnitzii. Front. Microbiol..

[10] Miquel S., Leclerc M., Martin R., Chain F., Lenoir M., Raguideau S., Hudault S., Bridonneau C., Northen T., Bowen B. (2015). Identification of metabolic signatures linked to anti-inflammatory effects of Faecalibacterium prausnitzii. mBio.

[11] Quévrain E., Maubert M.A., Michon C., Chain F., Marquant R., Tailhades J., Miquel S., Carlier L., Bermúdez-Humarán L.G., Pigneur B. (2016). Identification of an anti-inflammatory protein from *Faecalibacterium prausnitzii*, a commensal bacterium deficient in Crohn’s disease. Gut.

[12] Martín R., Miquel S., Chain F., Natividad J.M., Jury J., Lu J., Sokol H., Theodorou V., Bercik P., Verdu E.F. (2015). Faecalibacterium prausnitzii prevents physiological damages in a chronic low-grade inflammation murine model. BMC Microbiol..

[13] Hill C., Guarner F., Reid G., Gibson G.R., Merenstein D.J., Pot B., Morelli L., Canani R.B., Flint H.J., Salminen S. (2014). The International Scientific Association for Probiotics and Prebiotics consensus statement on the scope and appropriate use of the term probiotic. Nat. Rev. Gastroenterol. Hepatol..

[14] Palleja A., Mikkelsen K.H., Forslund S.K., Kashani A., Allin K.H., Nielsen T., Hansen T.H., Liang S., Feng Q., Zhang C. (2018). Recovery of gut microbiota of healthy adults following antibiotic exposure. Nat. Microbiol..

[15] David L.A., Maurice C.F., Carmody R.N., Gootenberg D.B., Button J.E., Wolfe B.E., Ling A.V., Devlin A.S., Varma Y., Fischbach M.A. (2014). Diet rapidly and reproducibly alters the human gut microbiome. Nature.

[16] Mardinoglu A., Wu H., Bjornson E., Zhang C., Hakkarainen A., Räsänen S.M., Lee S., Mancina R.M., Bergentall M., Pietiläinen K.H. (2018). An Integrated Understanding of the Rapid Metabolic Benefits of a Carbohydrate-Restricted Diet on Hepatic Steatosis in Humans. Cell Metab..

[17] Ruiz-Saavedra S., Salazar N., Suárez A., de Los Reyes-Gavilán C.G., Gueimonde M., González S. (2020). Comparison of Different Dietary Indices as Predictors of Inflammation, Oxidative Stress and Intestinal Microbiota in Middle-Aged and Elderly Subjects. Nutrients.

[18] Dewulf E.M., Cani P.D., Claus S.P., Fuentes S., Puylaert P.G., Neyrinck A.M., Bindels L.B., de Vos W.M., Gibson G.R., Thissen J.P. (2013). Insight into the prebiotic concept: Lessons from an exploratory, double blind intervention study with inulin-type fructans in obese women. Gut.

[19] Hustoft T.N., Hausken T., Ystad S.O., Valeur J., Brokstad K., Hatlebakk J.G., Lied G.A. (2017). Effects of varying dietary content of fermentable short-chain carbohydrates on symptoms, fecal microenvironment, and cytokine profiles in patients with irritable bowel syndrome. Neurogastroenterol. Motil. Off. J. Eur. Gastrointest. Motil. Soc..

[20] Ramirez-Farias C., Slezak K., Fuller Z., Duncan A., Holtrop G., Louis P. (2009). Effect of inulin on the human gut microbiota: Stimulation of Bifidobacterium adolescentis and Faecalibacterium prausnitzii. Br. J. Nutr..

[21] Fernando W.M., Hill J.E., Zello G.A., Tyler R.T., Dahl W.J., Van Kessel A.G. (2010). Diets supplemented with chickpea or its main oligosaccharide component raffinose modify faecal microbial composition in healthy adults. Benef. Microbes.

[22] Hooda S., Boler B.M., Serao M.C., Brulc J.M., Staeger M.A., Boileau T.W., Dowd S.E., Fahey G.C., Swanson K.S. (2012). 454 pyrosequencing reveals a shift in fecal microbiota of healthy adult men consuming polydextrose or soluble corn fiber. J. Nutr..

[23] McDonald D., Hyde E., Debelius J.W., Morton J.T., Gonzalez A., Ackermann G., Aksenov A.A., Behsaz B., Brennan C., Chen Y. (2018). American Gut: An Open Platform for Citizen Science Microbiome Research. mSystems.

[24] Gonzalez A., Navas-Molina J.A., Kosciolek T., McDonald D., Vázquez-Baeza Y., Ackermann G., DeReus J., Janssen S., Swafford A.D., Orchanian S.B. (2018). Qiita: Rapid, web-enabled microbiome meta-analysis. Nat. Methods.

[25] Amir A., McDonald D., Navas-Molina J.A., Kopylova E., Morton J.T., Zech Xu Z., Kightley E.P., Thompson L.R., Hyde E.R., Gonzalez A. (2017). Deblur Rapidly Resolves Single-Nucleotide Community Sequence Patterns. mSystems.

[26] Caporaso J.G., Kuczynski J., Stombaugh J., Bittinger K., Bushman F.D., Costello E.K., Fierer N., Peña A.G., Goodrich J.K., Gordon J.I. (2010). QIIME allows analysis of high-throughput community sequencing data. Nat. Methods.

[27] Pedregosa F., Varoquaux G., Gramfort A., Michel V., Thirion B., Grisel O., Blondel M., Prettenhofer P., Weiss R., Dubourg V. (2011). Scikit-learn: Machine Learning in Python. JMLR.

[28] Duncan S.H., Hold G.L., Harmsen H.J.M., Stewart C.S., Flint H.J. (2002). Growth requirements and fermentation products of Fusobacterium prausnitzii, and a proposal to reclassify it as *Faecalibacterium prausnitzii* gen. nov. comb. nov. Int. J. Syst. Evol. Microbiol..

[29] Van den Abbeele P., Taminiau B., Pinheiro I., Duysburgh C., Jacobs H., Pijls L., Marzorati M. (2018). Arabinoxylo-Oligosaccharides and Inulin Impact Inter-Individual Variation on Microbial Metabolism and Composition, Which Immunomodulates Human Cells. J. Agric. Food Chem..

[30] Pérez-Burillo S., Molino S., Navajas-Porras B., Valverde-Moya Á.J., Hinojosa-Nogueira D., López-Maldonado A., Pastoriza S., Rufián-Henares J.Á. (2021). An in vitro batch fermentation protocol for studying the contribution of food to gut microbiota composition and functionality. Nat. Protoc..

[31] Nadkarni M.A., Martin F.E., Jacques N.A., Hunter N. (2002). Determination of bacterial load by real-time PCR using a broad-range (universal) probe and primers set. Microbiology.

[32] Lopez-Siles M., Martinez-Medina M., Busquets D., Sabat-Mir M., Duncan S.H., Flint H.J., Aldeguer X., Garcia-Gil L.J. (2014). Mucosa-associated Faecalibacterium prausnitzii and Escherichia coli co-abundance can distinguish Irritable Bowel Syndrome and Inflammatory Bowel Disease phenotypes. Int. J. Med. Microbiol..

[33] Wishart D.S., Guo A., Oler E., Wang F., Anjum A., Peters H., Dizon R., Sayeeda Z., Tian S., Lee B.L. (2021). HMDB 5.0: The Human Metabolome Database for 2022. Nucleic Acids Res..

[34] R Core Team (2021). R: A Language and Environment for Statistical Computing.

[35] Madrid-Gambin F., Oller-Moreno S., Fernandez L., Bartova S., Giner M.P., Joyce C., Ferraro F., Montoliu I., Moco S., Marco S. (2020). AlpsNMR: An R package for signal processing of fully untargeted NMR-based metabolomics. Bioinformatics.

[36] Lundberg S.M., Erion G., Chen H., DeGrave A., Prutkin J.M., Nair B., Katz R., Himmelfarb J., Bansal N., Lee S.-I. (2020). From local explanations to global understanding with explainable AI for trees. Nat. Mach. Intell..

[37] Lopez-Siles M., Khan T.M., Duncan S.H., Harmsen H.J.M., Garcia-Gil L.J., Flint H.J. (2012). Cultured Representatives of Two Major Phylogroups of Human Colonic *Faecalibacterium prausnitzii* Can Utilize Pectin, Uronic Acids, and Host-Derived Substrates for Growth. Appl. Environ. Microbiol..

[38] Fitzgerald C.B., Shkoporov A.N., Sutton T.D.S., Chaplin A.V., Velayudhan V., Ross R.P., Hill C. (2018). Comparative analysis of Faecalibacterium prausnitzii genomes shows a high level of genome plasticity and warrants separation into new species-level taxa. BMC Genom..

[39] Ruiz-Ojeda F.J., Plaza-Díaz J., Sáez-Lara M.J., Gil A. (2019). Effects of Sweeteners on the Gut Microbiota: A Review of Experimental Studies and Clinical Trials. Adv. Nutr..

[40] Fernández-Bañares F. (2022). Carbohydrate Maldigestion and Intolerance. Nutrients.

[41] Soto-Martin E.C., Warnke I., Farquharson F.M., Christodoulou M., Horgan G., Derrien M., Faurie J.-M., Flint H.J., Duncan S.H., Louis P. (2020). Vitamin Biosynthesis by Human Gut Butyrate-Producing Bacteria and Cross-Feeding in Synthetic Microbial Communities. mBio.

[42] Vital M., Karch A., Pieper D.H. (2017). Colonic Butyrate-Producing Communities in Humans: An Overview Using Omics Data. mSystems.

[43] Cotillard A., Cartier-Meheust A., Litwin N.S., Chaumont S., Saccareau M., Lejzerowicz F., Tap J., Koutnikova H., Lopez D.G., McDonald D. (2021). A posteriori dietary patterns better explain variations of the gut microbiome than individual markers in the American Gut Project. Am. J. Clin. Nutr..

[44] Cuesta-Zuluaga J.d.l., Kelley S.T., Chen Y., Escobar J.S., Mueller N.T., Ley R.E., McDonald D., Huang S., Swafford A.D., Knight R. (2019). Age- and Sex-Dependent Patterns of Gut Microbial Diversity in Human Adults. mSystems.

[45] Taylor B.C., Lejzerowicz F., Poirel M., Shaffer J.P., Jiang L., Aksenov A., Litwin N., Humphrey G., Martino C., Miller-Montgomery S. (2020). Consumption of Fermented Foods Is Associated with Systematic Differences in the Gut Microbiome and Metabolome. mSystems.

[46] Zhu C., Wang X., Li J., Jiang R., Chen H., Chen T., Yang Y. (2022). Determine independent gut microbiota-diseases association by eliminating the effects of human lifestyle factors. BMC Microbiol..

[47] Ullmann T., Peschel S., Finger P., Müller C.L., Boulesteix A.-L. (2023). Over-optimism in unsupervised microbiome analysis: Insights from network learning and clustering. PLoS Comput. Biol..

[48] Moreno-Indias I., Sánchez-Alcoholado L., Pérez-Martínez P., Andrés-Lacueva C., Cardona F., Tinahones F., Queipo-Ortuño M.I. (2016). Red wine polyphenols modulate fecal microbiota and reduce markers of the metabolic syndrome in obese patients. Food Funct..

[49] Clements R.S., Darnell B. (1980). Myo-inositol content of common foods: Development of a high-myo-inositol diet. Am. J. Clin. Nutr..

[50] Jiang Z., Sun T.-y., He Y., Gou W., Zuo L.-s.-y., Fu Y., Miao Z., Shuai M., Xu F., Xiao C. (2020). Dietary fruit and vegetable intake, gut microbiota, and type 2 diabetes: Results from two large human cohort studies. BMC Med..

[51] van Soest A.P.M., Hermes G.D.A., Berendsen A.A.M., van de Rest O., Zoetendal E.G., Fuentes S., Santoro A., Franceschi C., de Groot L.C.P.G.M., de Vos W.M. (2020). Associations between Pro- and Anti-Inflammatory Gastro-Intestinal Microbiota, Diet, and Cognitive Functioning in Dutch Healthy Older Adults: The NU-AGE Study. Nutrients.

[52] Sakamoto M., Sakurai N., Tanno H., Iino T., Ohkuma M., Endo A. (2022). Genome-based, phenotypic and chemotaxonomic classification of Faecalibacterium strains: Proposal of three novel species Faecalibacterium duncaniae sp. nov. Faecalibacterium hattorii sp. nov. and Faecalibacterium gallinarum sp. nov. Int. J. Syst. Evol. Microbiol..

[53] Koecher K.J., Noack J.A., Timm D.A., Klosterbuer A.S., Thomas W., Slavin J.L. (2014). Estimation and Interpretation of Fermentation in the Gut: Coupling Results from a 24 h Batch in Vitro System with Fecal Measurements from a Human Intervention Feeding Study Using Fructo-oligosaccharides, Inulin, Gum Acacia, and Pea Fiber. J. Agric. Food Chem..

[54] Scalera V., Natuzzi D., Prezioso G. (1991). myo-inositol transport in rat intestinal brush border membrane vesicles, and its inhibition by D-glucose. Biochim. Biophys. Acta.

[55] Bagheri S., Zolghadri S., Stanek A. (2022). Beneficial Effects of Anti-Inflammatory Diet in Modulating Gut Microbiota and Controlling Obesity. Nutrients.

[56] Kim H., Jeong Y., Kang S., You H.J., Ji G.E. (2020). Co-Culture with Bifidobacterium catenulatum Improves the Growth, Gut Colonization, and Butyrate Production of Faecalibacterium prausnitzii: In Vitro and In Vivo Studies. Microorganisms.

[57] Costabile A., Kolida S., Klinder A., Gietl E., Bäuerlein M., Frohberg C., Landschütze V., Gibson G.R. (2010). A double-blind, placebo-controlled, cross-over study to establish the bifidogenic effect of a very-long-chain inulin extracted from globe artichoke (*Cynara scolymus*) in healthy human subjects. Br. J. Nutr..

[58] Ramnani P., Gaudier E., Bingham M., van Bruggen P., Tuohy K.M., Gibson G.R. (2010). Prebiotic effect of fruit and vegetable shots containing Jerusalem artichoke inulin: A human intervention study. Br. J. Nutr..

[59] Dogra S. (2022). Systems and Methods for Estimating, from Food Frequency Questionnaire Based Nutrients Intake Data, The Relative Amounts of Faecalibacterium Prausnitzii (Fprau) in the Gut Microbiome Ecosystem and Associated Recommendations to Improve Faecalibacterium Prausnitzii. https://worldwide.espacenet.com/patent/search/family/075825630/publication/WO2022233924A1?q=pn%3DWO2022233924A1.

[60] Chou C., Dogra S., Dardinier A. (2022). Compositions and Methods Using at least One of Inositol, Erythritol or Sorbitol to Enhance Growth of Faecalibacterium Prausnitzii. https://worldwide.espacenet.com/patent/search/family/081941092/publication/WO2022233922A1?q=pn%3DWO2022233922A1.

