# Supplementary material for: Application of Computational Data Modeling to a Large-Scale Population Cohort Assists the Discovery of Inositol as a Strain-Specific Substrate for Faecalibacterium prausnitzii
Source: Nutrients. 2023 Mar 7;15(6):1311. doi: 10.3390/nu15061311 (PMC10051675; doi:10.3390/nu15061311)
Supplement: Supplementary file 1 [file nutrients-15-01311-s001.zip › nutrients-2199282-supplementary.pdf]

nutrients

Application of Computational Data Modeling to a Large-Scale Population Cohort Assists the Discovery of Inositol as a Strain-Specific Substrate for *Faecalibacterium prausnitzii*

Shaillay Kumar Dogra [1], Adrien Dardinier [1], Fabio Mainardi [2], Léa Siegwald [2], Simona Bartova [3], Caroline Le Roy [1] and Chieh Jason Chou [1,*]

[1] Department of Gastrointestinal Health, Nestlé Institute of Health Sciences, Nestlé Research, CH-1000 Lausanne, Switzerland
[2] Department of Data Sciences and Precision Nutrition, Nestlé Institute of Health Sciences, Nestlé Research, CH-1000 Lausanne, Switzerland
[3] Department of Bioanalytics, Nestlé Institute of Food Safety and Analytical Sciences, Nestlé Research, CH-1000 Lausanne, Switzerland
* Correspondence: chieh-jason.chou@rd.nestle.com; Tel.: +41-21-785-92-89

Supplementary Materials

Supplementary Table S1. Nutrients in the models.

Nutrient Names in the Models	Full Name	Unit
alcohol	Alcohol	g
inositol	Inositol	g
xylitol	Xylitol	g
sfa220	SFA 22:0 (behenic acid)	g
delttoco	Delta-Tocopherol	mg
lycopene	Lycopene	mcg
alphacar	Alpha-Carotene (provitamin A carotenoid)	mcg
galactos	Galactose	g
vita_re	Total Vitamin A Activity (Retinol Equivalents)	mcg
sucrose	Sucrose	g
betaine	Betaine	mg
betacryp	Beta-Cryptoxanthin (provitamin A carotenoid)	mcg
vita_rae	Total Vitamin A Activity (Retinol Activity Equivalents)	mcg
thiamin	Thiamin (vitamin B1)	mg
coumest	Coumestrol	mg
maltose	Maltose	g
vitd3	Vitamin D3 (cholecalciferol)	mcg
pfa183	PUFA 18:3 (linolenic acid)	g
choline	Choline	mg
vita_iu	Total Vitamin A Activity (International Units)	IU
erythr	Erythritol	g
tfa182t	TRANS 18:2 (trans-octadecadienoic acid [linolelaidic acid]); includes c-t, t-c, t-t)	g
tagatose	Tagatose	mg
ribofla	Riboflavin (vitamin B2)	mg
addsugar	Added Sugars (by Available Carbohydrate)	g
vitd_iu	Vitamin D (calciferol)	IU
vitd	Vitamin D (calciferol)	mcg
pfa225	PUFA 22:5 (docosapentaenoic acid [DPA])	g
sorbitol	Sorbitol	g
adsugtot	Added Sugars (by Total Sugars)	g

Supplementary Table S2. Performance of various models.

Model ID	Model Category	ML Algorithm	TARGET	Bin Definition	Target Label
A	low-high	RANDOM FOREST	cube_trnfrmd Fprau	Q1 vs Q4	low
B	low-not_low	RANDOM FOREST	cube_trnfrmd Fprau	Q1 vs Q2, Q3 and Q4	low
C	high-not_high	RANDOM FOREST	cube_trnfrmd Fprau	Q1, Q2, and Q3 vs Q4	high
D	low-high	RANDOM FOREST	cube_trnfrmd Fprau	< mean - 1SD vs > mean + 1SD	low
E	low-not_low	RANDOM FOREST	cube_trnfrmd Fprau	< mean - 1SD vs rest	low
F	high-not_high	RANDOM FOREST	cube_trnfrmd Fprau	> mean + 1SD vs rest	high
G	low-high	XGBOOST	cube_trnfrmd Fprau	< mean - 1SD vs > mean + 1SD	low
H	low-not_low	XGBOOST	cube_trnfrmd Fprau	< mean - 1SD vs rest	low
I	high-not_high	XGBOOST	cube_trnfrmd Fprau	> mean + 1SD vs rest	high

Model ID	train_ROC AUC	train_PR AUC	Train (*n*)	test_ROC AUC	test_PR AUC	Test (*n*)
A	0.63 ± 0.01	0.66 ± 0.02	1532	0.61	0.65	386
B	0.64 ± 0.01	0.41 ± 0.02	1554	0.64	0.41	764
C	0.58 ± 0.02	0.3 ± 0.02	1532	0.61	0.32	764
D	0.66 ± 0.03	0.71 ± 0.03	828	0.64	0.71	216
E	0.65 ± 0.02	0.3 ± 0.03	896	0.68	0.33	764
F	0.56 ± 0.02	0.16 ± 0.02	828	0.58	0.17	764
G	0.65 ± 0.03	0.7 ± 0.03	828	0.65	0.73	216
H	0.66 ± 0.03	0.29 ± 0.03	896	0.65	0.32	764
I	0.56 ± 0.02	0.16 ± 0.02	828	0.58	0.17	764

Supplementary Table S3. Summary of metadata of a subset of American Gut Project participants used in the study.

Summary of metadata of a subset of American Gut Project participants used in the study	
	**Overall (*n* = 3816)**
**sex**	
female	2268 (59.4%)
male	1486 (38.9%)
other	8 (0.2%)
unspecified	54 (1.4%)
**age_cat**	
20s	261 (6.8%)
30s	520 (13.6%)
40s	725 (19.0%)
50s	833 (21.8%)
60s	1020 (26.7%)
70+	253 (6.6%)
baby	14 (0.4%)
child	49 (1.3%)
teen	36 (0.9%)
Unspecified	105 (2.8%)
**host_age**	
Mean (SD)	51.3 (15.6)
Median [Min, Max]	53.0 [0.100, 173]
Missing	104 (2.7%)
**host_height**	
Mean (SD)	174 (67.3)
Median [Min, Max]	170 [12.7, 1800]
Missing	39 (1.0%)
**host_weight**	
Mean (SD)	71.1 (17.9)
Median [Min, Max]	69.0 [0.454, 214]
Missing	37 (1.0%)
**host_body_mass_index**	
Mean (SD)	31.3 (182)
Median [Min, Max]	23.6 [0.200, 6890]
Missing	53 (1.4%)
**bmi_cat**	
Normal	2094 (54.9%)
Obese	364 (9.5%)
Overweight	1103 (28.9%)

Underweight	167 (4.4%)
Unspecified	88 (2.3%)
**race**	
African American	20 (0.5%)
Asian or Pacific Islander	108 (2.8%)
Caucasian	3437 (90.1%)
Hispanic	75 (2.0%)
Other	106 (2.8%)
Unspecified	70 (1.8%)
**country_residence**	
Australia	56 (1.5%)
Austria	4 (0.1%)
Belgium	6 (0.2%)
Brazil	1 (0.0%)
Canada	33 (0.9%)
China	2 (0.1%)
Czech Republic	4 (0.1%)
Denmark	5 (0.1%)
Estonia	1 (0.0%)
France	12 (0.3%)
Germany	18 (0.5%)
Greece	2 (0.1%)
Guernsey	1 (0.0%)
Hong Kong	1 (0.0%)
Ireland	17 (0.4%)
Italy	7 (0.2%)
Japan	2 (0.1%)
Jersey	2 (0.1%)
Mexico	3 (0.1%)
Netherlands	4 (0.1%)
New Zealand	3 (0.1%)
Norway	2 (0.1%)
Saudi Arabia	1 (0.0%)
Serbia	1 (0.0%)
Singapore	1 (0.0%)
Slovakia	4 (0.1%)
Spain	6 (0.2%)
Sweden	11 (0.3%)
Switzerland	15 (0.4%)
United Arab Emirates	4 (0.1%)
United Kingdom	829 (21.7%)
United States	1654 (43.3%)
United States Minor Outlying	1 (0.0%)

Islands	
Unspecified	1103 (28.9%)
**diet_type**	
Omnivore	2929 (76.8%)
Omnivore but do not eat red meat	247 (6.5%)
Unspecified	97 (2.5%)
Vegan	121 (3.2%)
Vegetarian	185 (4.8%)
Vegetarian but eat seafood	237 (6.2%)
**exercise_frequency**	
Daily	811 (21.3%)
Never	102 (2.7%)
Occasionally (1-2 times/week)	1040 (27.3%)
Rarely (a few times/month)	392 (10.3%)
Regularly (3-5 times/week)	1397 (36.6%)
Unspecified	74 (1.9%)

Supplementary Table S4. Mean intake of nutrients that are significantly different between the Low and notLow *F. prausnitzii* categories.

Mean intake of nutrients that are significantly different between the low and notlow *F. prausnitzii* categories				
nutrient	low (*n* = 560)	notlow (*n* = 3256)	*p*.value	*p*.adj
alcohol	9,31	13,72	0	0
inositol	1696,51	1944,21	0	0
aspartam	45,62	23,45	0	0
betacryp	10107,05	10987,59	0	0
betacar	159,3	175,29	0	0,01
vita_iu	0,14	0,16	0	0,01
vita_re	8240,81	8824,92	0	0,01
alphacar	4,28	4,56	0	0,02
pectins	19797,68	21525,04	0	0,02
vita_rae	1341,38	1437,73	0	0,02
lutzeax	2260,96	2441,68	0	0,04

Wilcoxon rank sum test was used to compare the two groups and Benjamini-Hochsberg method was applied to control the false discovery rate. Only significant differences are shown (adjusted $p<0.05$).

Supplementary Table S5. Normalized mean intake (2000 kcal) of nutrients that are significantly different between the Low and notLow *F. prausnitzii* categories.

Normalized mean intake (2000 kcal) of nutrients that are significantly different between the low and notlow F. *prausnitzii* categories				
nutrient	low (*n* = 560)	notlow (*n* = 3256)	*p*.value	*p*.adj
alcohol	9,87	14,75	0	0
inositol	2040,82	2284,09	0	0,0000028
proline	48,44	25,9	0,0000064	0,000309
aspartam	186,62	203,74	0,0000105	0,0003791
glutamic	2,37	2,24	0,000045	0,0013057
betacryp	11,1	10,6	0,0002905	0,0070194
delttoco	15,77	14,98	0,0005321	0,0110212
gammtoco	0,16	0,18	0,0018005	0,0290081
thiamin	5,13	4,8	0,001611	0,0290081
selenium	119,48	114,55	0,0020092	0,0291329
alphacar	1,54	1,46	0,0026063	0,0343557

Wilcoxon rank sum test was used to compare the two groups and Benjamini-Hochsberg method was applied to control the false discovery rate. Only significant differences are shown (adjusted $p<0.05$).

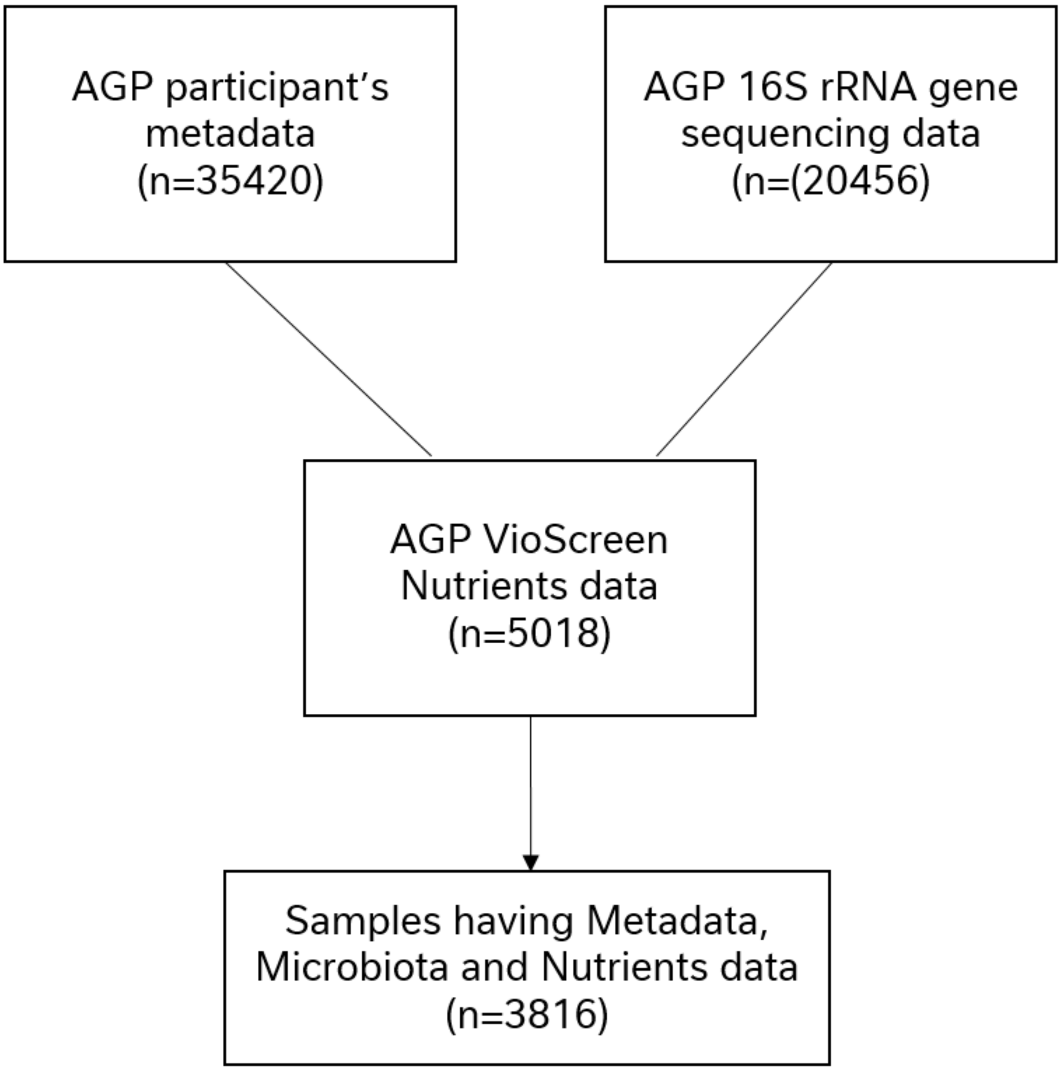

Supplementary Figure S1. Selection of subjects for the analyses. American Gut Project (AGP) Metadata (*n*=35,420), 16S rRNA gene sequencing Microbiota data (*n*=20,456) and FFQ-based Vioscreen-derived Nutrients data (*n*=5,018) were downloaded from the respective repositories described in Methods and Materials. Only the subjects having all three data types available were included in the modeling analysis (*n*=3,816).

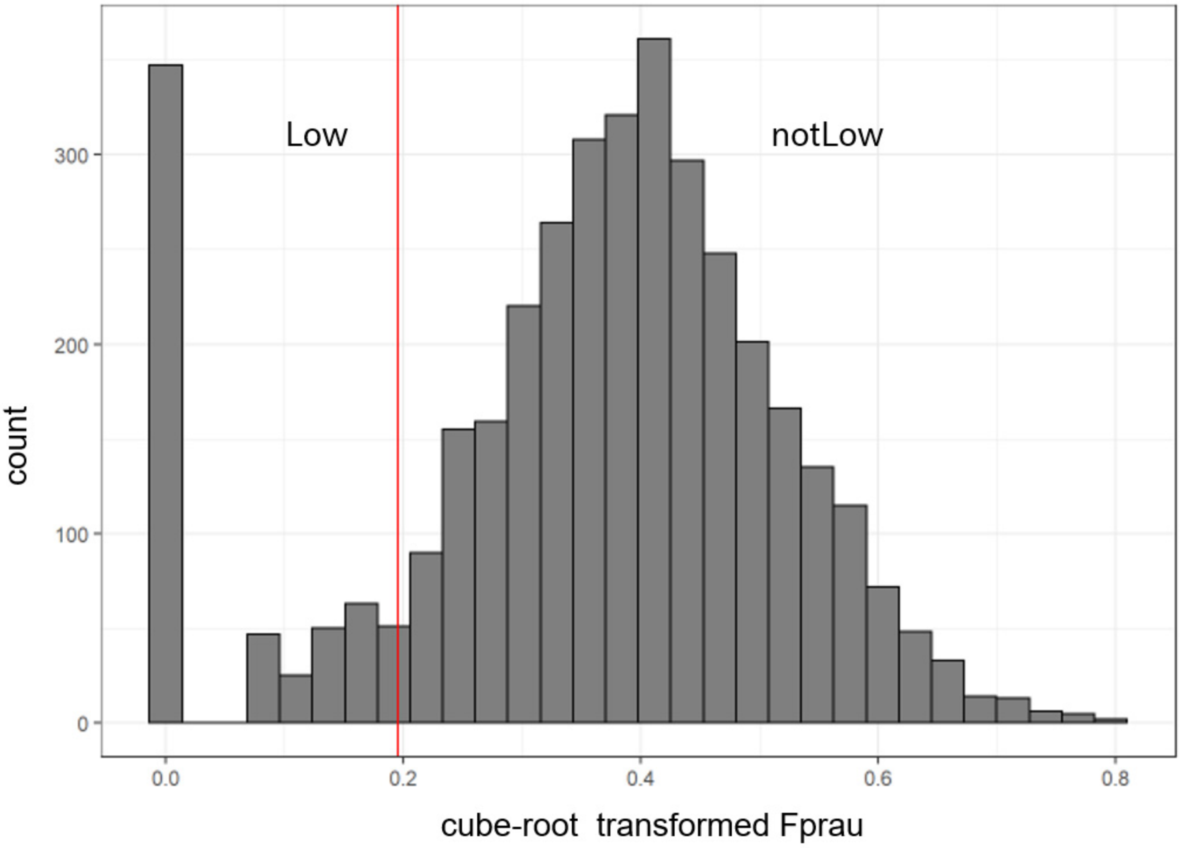

Supplementary Figure S2. Distribution of *F. prausnitzii* in the AGP sub-cohort. A subset of AGP subjects (*n*=3816) was selected with available metadata, FFQ data and 16S data. The relative abundance of *F. prausnitzii* was cube-transformed and the frequency distribution is shown. A normalized distribution was then used to create different categories (i.e. low vs notlow, high vs nothigh and low vs high).

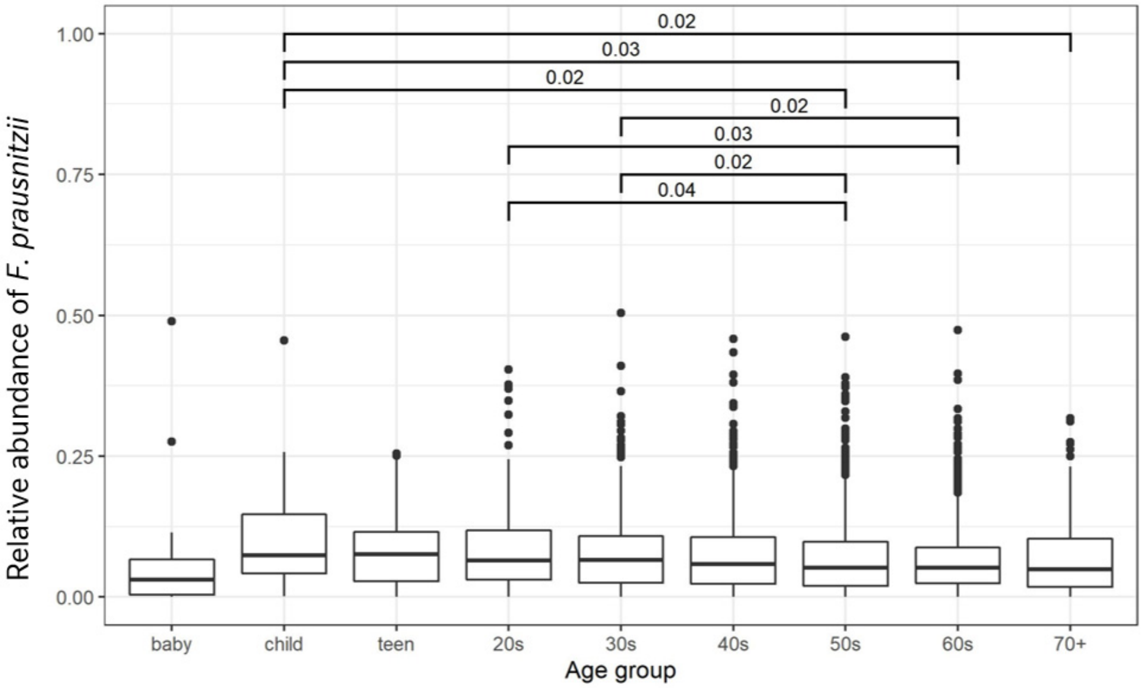

Supplementary Figure S3. Age-associated relationship with *F. prausnitzii* in AGP sub-cohort. Relationship between the relative abundance of *F. prausnitzii* and self-reported age of 3816 AGP subjects. Differences in *F. prausnitzii* relative abundance between age categories are shown. Statistical comparisons were performed with Kruskal-Wallis rank sum test followed by post hoc Dunn test. *p* values of statistically significant differences are shown on the top of cross bars.

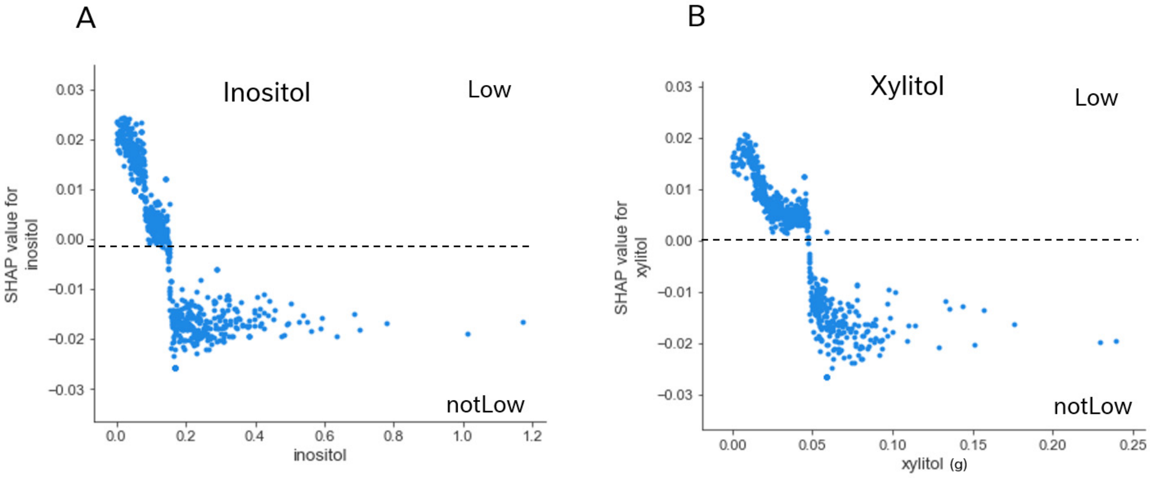

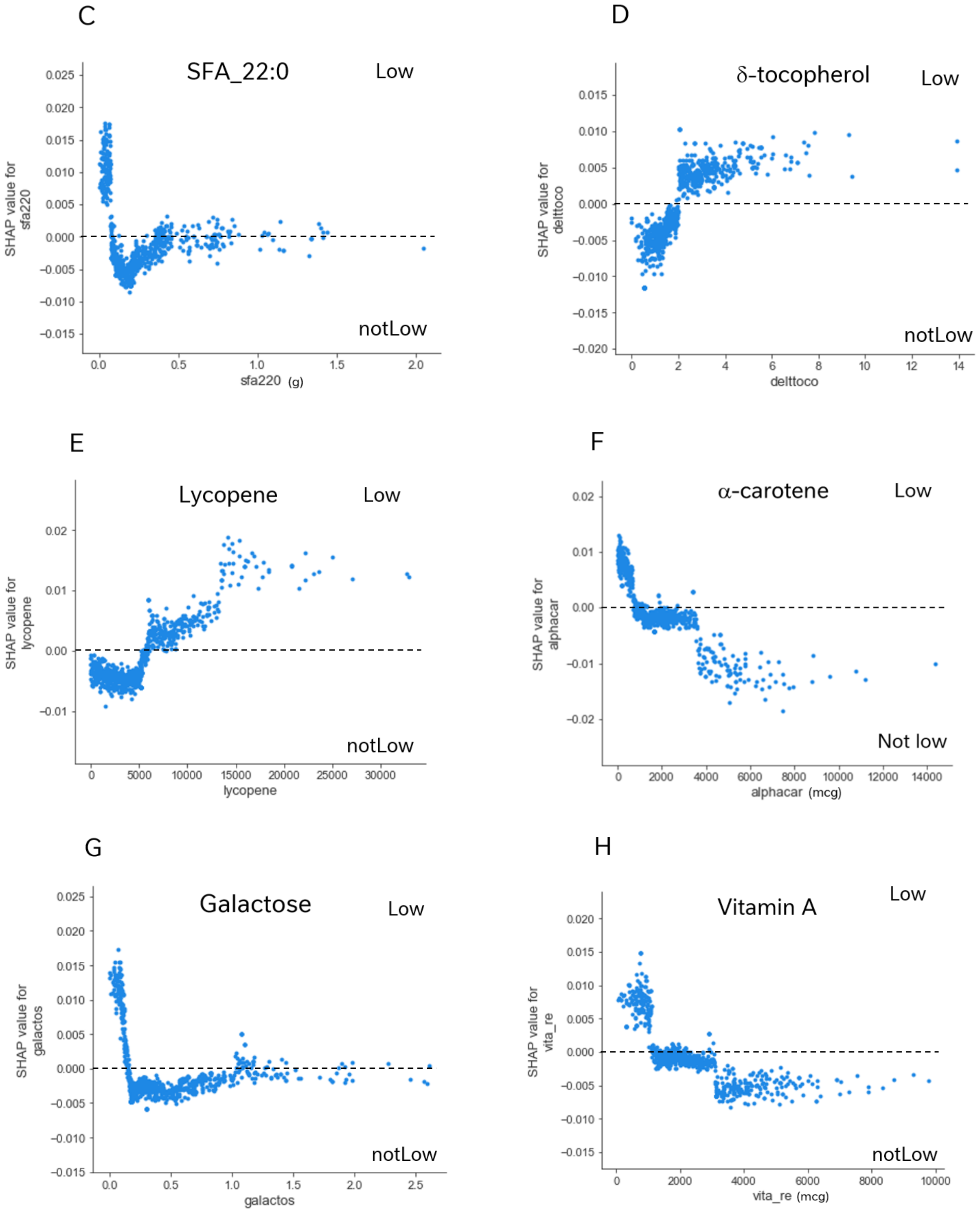
C
SFA_22:0
Low
notLow
SHAP value for sfa220
sfa220 (g)
D
δ-tocopherol
Low
notLow
SHAP value for delttoco
delttoco
E
Lycopene
Low
notLow
SHAP value for lycopene
lycopene
F
α-carotene
Low
Not low
SHAP value for alphacar
alphacar (mcg)
G
Galactose
Low
notLow
SHAP value for galactos
galactos
H
Vitamin A
Low
notLow
SHAP value for vita_re
vita_re (mcg)

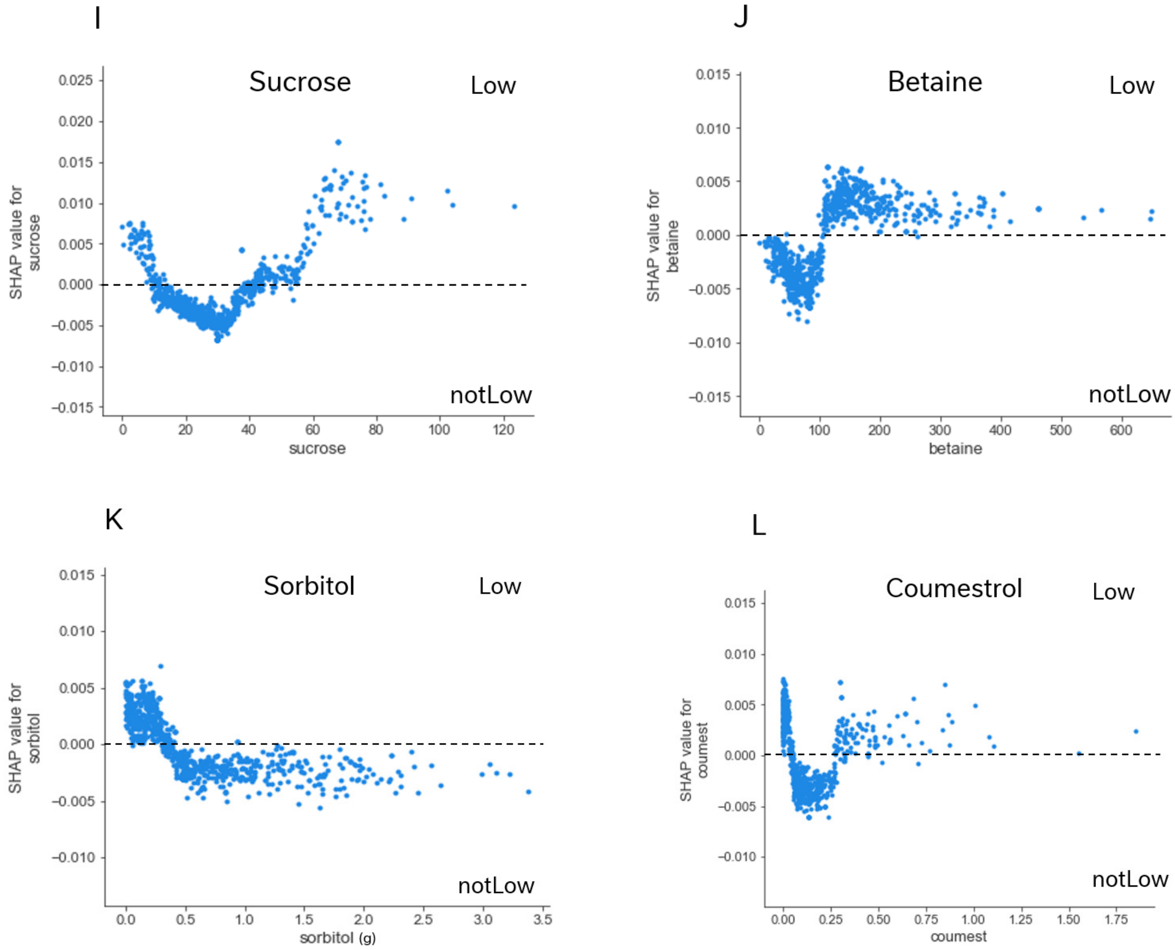

Supplementary Figure S4. SHAP Dependence plots of top nutrients in the model. SHAP dependence plots of top 12 nutrients except for alcohol are presented in (**A**, inositol); (**B**, xylitol); (**C**, SFA_22:0); (**D**, d-tocopherol); (**E**, lycopene); (**F**, a-carotene); (**G**, galactose); (**H**, vita_re); (**I**, sucrose); (**J**, betaine); (**K**, sorbitol); (**L**, coumest). For each nutrient feature, the SHAP Dependence plot shows the intake value on the x-axis and the corresponding Shapley value on the y-axis. The reference class here was "Low". Thus, the positive coefficients of SHAP value on the y-axes, with the corresponding x-values, indicate how the model was affected in predicting the "Low" class using this feature. For example, as shown in Supporting figure 1A, specific intake values of inositol had a relation with impact on model output. Low inositol intake has the *F. prausnitzii* status in the "Low" class, while higher intake amounts of inositol have the Fprau status in "notLow" class. When interpreting, please note that the final prediction of the model is result of a complex multivariate analysis. Thus, the final impact on the *F. prausnitzii* status of an individual, e.g., as Low or notLow, was a combination of different features to one single output from the model.

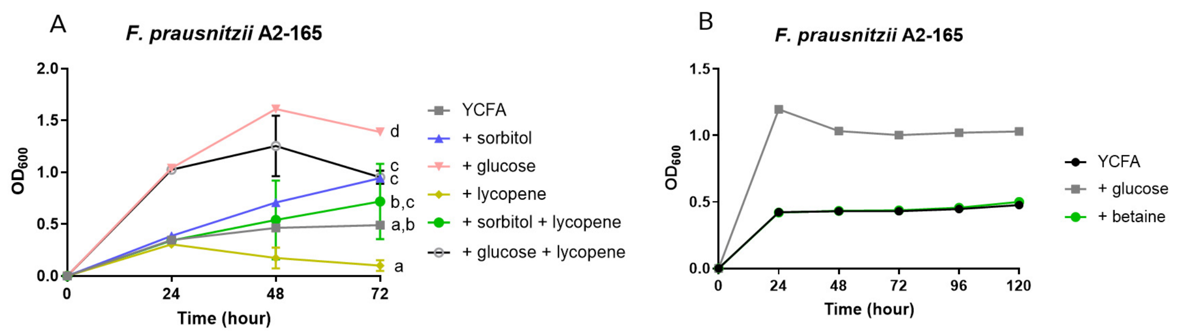

Supplementary Figure S5. The growth of *F. prausnitzii* in single or combination of nutrients. Anaerobic culture conditions of *F. prausnitzii* A2-165 are described in Methods and Materials. The growth of the bacteria over time was measure with optical density (OD_{600}). Response of *F. prausnitzii* to glucose, sorbitol, lycopene, sorbitol+lycopene and glucose+lycopene are shown (**A**). Growth of the bacteria under glucose or betaine is shown in (**B**). Results are mean ± SEM, *n* = 3. Statistical analysis was performed with ANOVA followed by Tukey post hoc analysis. When treatment groups share a same letter, it means no statistical difference between the groups.

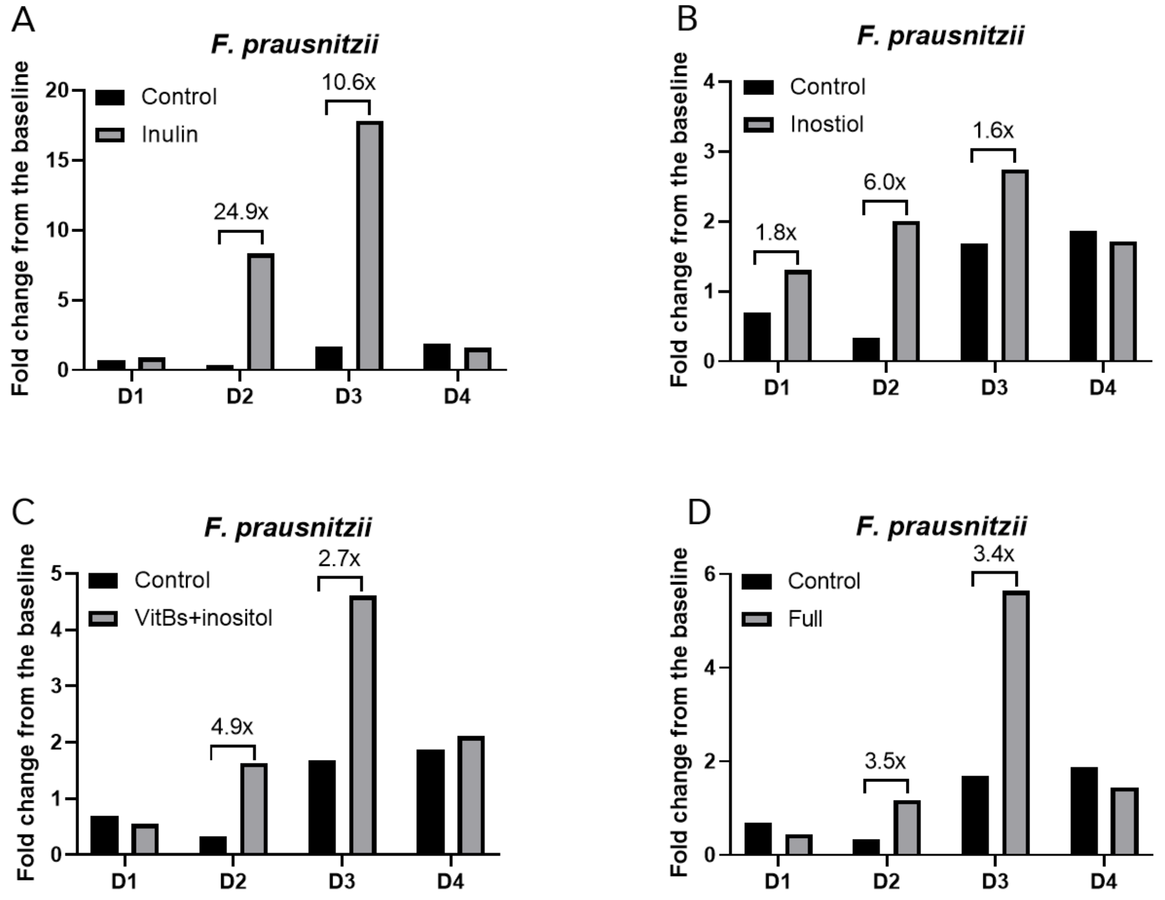

Supplementary Figure S6. Effect of nutrients on the growth of *F. prausnitzii* in a complex community is donor dependent. The effect of single or combination of nutrients on number of *F. prausnitzii* in a mix community was examined in an in vitro batch fermentation system as described in Methods and Materials. The number of *F. prausnitzii* was quantified by qPCR using *F. pruastnizii* specific primers. The results of inulin (**A**), inositol (**B**), vitBs+inositol (**C**) and full (**D**) are shown. vitBs consists of vitamin B5, B6 and B12, and full denotes Vitamin A, vitamin B5, vitamin B6, vitamin B12, vitamin D. Fold change of *F. prausnitzii* at 24h from the baseline are shown for each of the four microbiome backgrounds and the differences between treatment and control are indicated when the number is larger than 1.5.

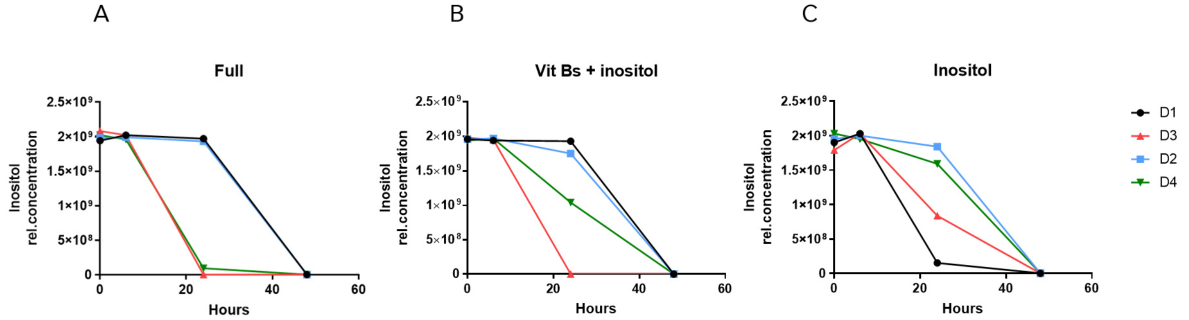

Supplementary Figure S7. Decrease of inositol signal over the duration of *in vitro* fermentation. Signal of inositol was extracted from metabolomic data and strength of the signal at each time point is shown. Results of full mix (**A**), vitB+inositol (**B**) and inositol alone (**C**) are shown. Color of line represents different microbiota background (D1-D4).

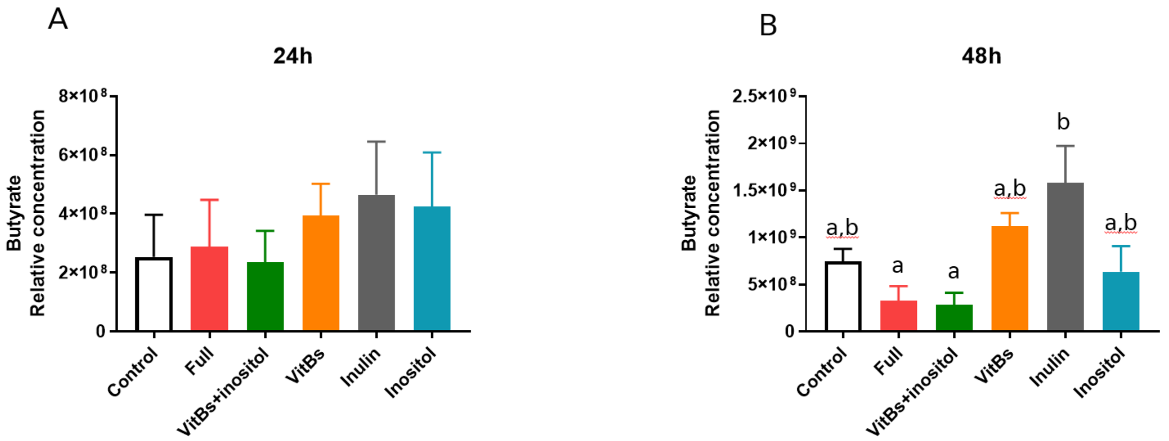

Supplementary Figure S8. Evaluation of nutrients and nutrient combinations on the levels of butyrate in batch fermentation experiments extracted from ^{1}H NMR metabolomics. Details of *in vitro* fermentation experiments are described in Methods and Materials. The relative concentrations of butyrate at 24h (**A**) and 48h (**B**) showed as a mean of 4 fecal donors. Results are mean ± SEM, n = 3. Statistical analysis was performed with ANOVA followed by Tukey post hoc analysis. Statistically significant difference among means was found at 48 h (p=0.0054), which was cause by increased levels of butyrate with inulin as indicated by multiple comparisons test. When treatment groups share a same letter, it means no statistical difference between the groups.

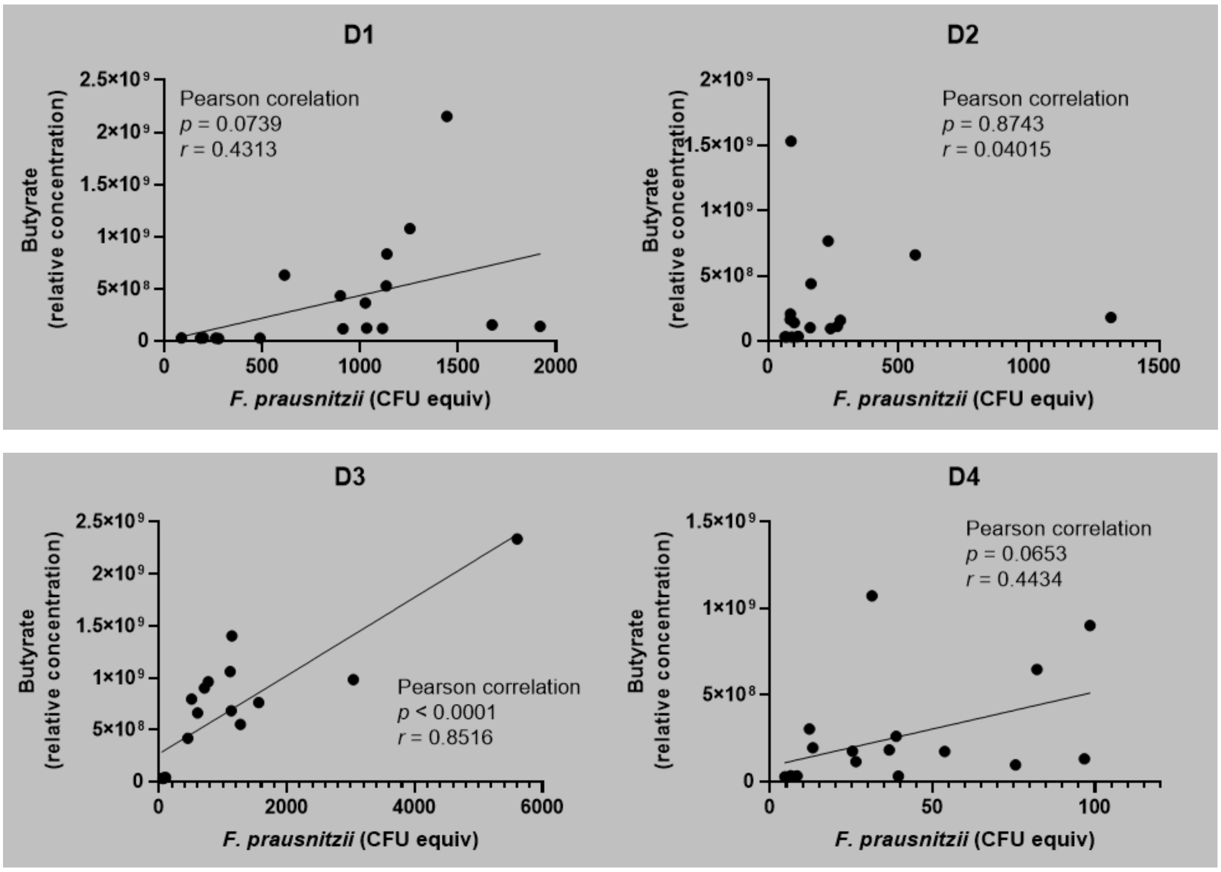

Supplementary Figure S9. Relationship between the number of *F. prausnitzii* and butyrate signals in the fermentation media. *F. prausnitzii* number was measured with *F. prausnitzii* specific qPCR, and butyrate signals were extracted from ^{1}HNMR spectra. Results include all time points (0, 6, 24 and 48h) and are presented according to the microbiota background, donor 1 (A), donor 2 (B), donor 3 (C) and donor 4 (D). Each (•) corresponds to a sample taken from the *in vitro* fermentation experiment. Pearson correlation analysis was performed to analyze the relationship between *F. prausnitzii* number and butyrate. *p*-values and correlation coefficients are shown in the insert of each panel.
